# Recent trends in bioartificial muscle engineering and their applications in cultured meat, biorobotic systems and biohybrid implants

**DOI:** 10.1038/s42003-022-03593-5

**Published:** 2022-07-22

**Authors:** Eva Schätzlein, Andreas Blaeser

**Affiliations:** 1 Technical University of Darmstadt, Institute for BioMedical Printing Technology, Darmstadt, Germany; 2 Technical University of Darmstadt, Centre for Synthetic Biology, Darmstadt, Germany

**Keywords:** Tissues, Tissue engineering

## Abstract

Recent advances in tissue engineering and biofabrication technology have yielded a plethora of biological tissues. Among these, engineering of bioartificial muscle stands out for its exceptional versatility and its wide range of applications. From the food industry to the technology sector and medicine, the development of this tissue has the potential to affect many different industries at once. However, to date, the biofabrication of cultured meat, biorobotic systems, and bioartificial muscle implants are still considered in isolation by individual peer groups. To establish common ground and share advances, this review outlines application-specific requirements for muscle tissue generation and provides a comprehensive overview of commonly used biofabrication strategies and current application trends. By solving the individual challenges and merging various expertise, synergetic leaps of innovation that inspire each other can be expected in all three industries in the future.

## Abstract (web summary)

This review discusses the recent advances in the engineering of bioartificial muscle. The applications highlighted include cultured meat, biorobotics, and muscle implants.

## Introduction

In the recent decade, the artificial biofabrication of muscle tissue attracted great interest. Due to its diverse applicability, for example as cultured meat^1^, biorobotic systems^2^, biohybrid implants in regenerative medicine^3^, or in disease modeling^4^, the popularity of this field of research steadily increased.

**Fig. 1:**
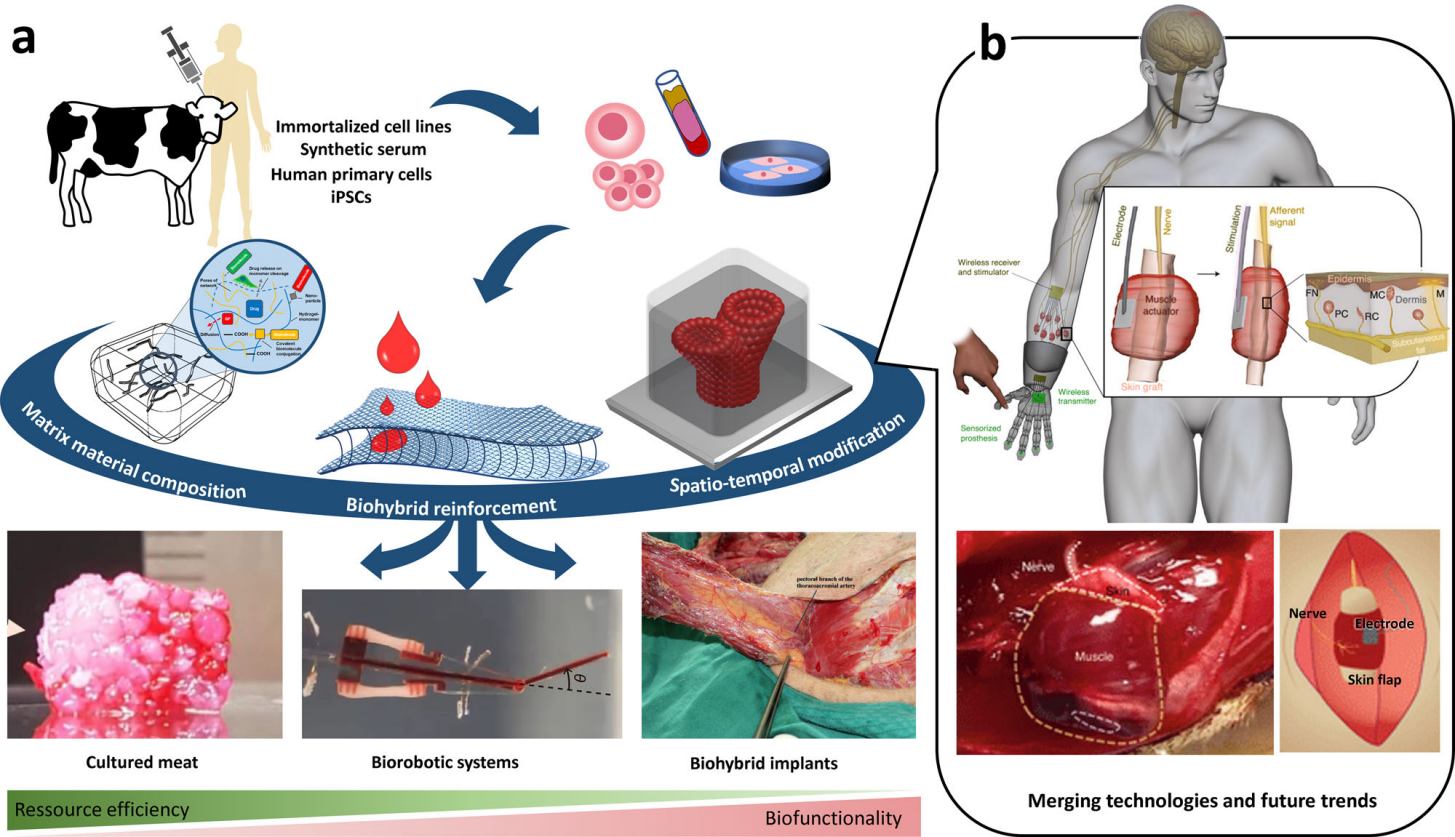
Biofabrication of muscle tissue and merging the expertise of the different fields of applications. Biofabrication of muscle tissue enables multiple applications (**a**) ranging from cultured meat^155^ (assembly of fibrous muscle, fat, and vascular tissues to cultured steak by Kang et al (CC BY 4.0)), over biorobotic systems (from ref. ^2^. Reprinted with permission from AAAS.) to biohybrid implants^3^ (the pectoral branch of the thoracoacromial artery was identified beneath the pectoralis major by Liu et al (CC BY 4.0)). This review provides a comprehensive overview on the most important cellular and material-specific requirements as well as dedicated biofabrication strategies(adapted from refs. ^22,148^ (Schematic illustration of the concept, experimental procedure, goal, and outlook of the study by Schäfer et al. (CC BY 4.0))) for each of the three fields of application. While biofabrication of cultured meat, biorobotic systems, and bioartificial muscle implants has mostly been studied in isolation so far, the technological fusion will unleash unexpected innovations and determine future trends. The recently published combination of biorobotic systems and biohybrid implants is a path-breaking pointer to what lies ahead (**b**, adapted from Srinivasan and co-workers^153^ (reprinted with permission from Springer Nature Limited: Nature Biomedical Engineering, A cutaneous mechanoneural interface for neuroprosthetic feedback, Srinivasan et al., Copyright 2021).

This article provides a comprehensive overview on recent biofabrication strategies dedicated to the production of muscle tissue for the first three fields of application (Fig. 1a). Latest success stories will be highlighted and critically discussed.

Muscle is a vascularized and innervated tissue composed of 90 % muscle cells, like myoblast and satellite cells and 10% fibroblasts and adipocytes^5^. While fibroblasts and adipocytes occur in bulk, muscle cells are composed of a hierarchical structure with aligned myofibers formed in a maturation process from fused elongated multinucleated myotubes influenced by chemical, mechanical and electrical cues^6,7^. Skeletal muscle tissue is embedded in an extracellular matrix (ECM) consisting mainly of collagen (Col) type I and III^8,9^. The artificial production of muscle tissue follows the generally known biofabrication process, comprising of three steps: preprocessing, manufacturing, and maturation^10^. First, the three-dimensional (3D) design and the required scaffold materials are defined. Next, cells and materials are assembled to form the tissue precursor. Commonly known biofabrication methods, such as cell seeding^11,12^, hydrogel casting^13,14^, or 3D-bioprinting^15–17^, are routinely applied. Ultimately, the tissue precursor undergoes a maturation phase. In muscle fabrication, this step is of particular relevance, since it determines the biological and mechanical functionality of the tissue^6,7,18^.

This review illustrates the diverse fields of muscle tissue fabrication and highlights what specifications and what strategies need to be considered for its application as cultured meat, biorobotic systems, and muscle implants. Finally, opportunities to further boost developments in muscle tissue engineering are elucidated.

## Fabrication strategies

Biofabrication faces various hurdles when recreating the microstructure and biological properties of muscle tissue. Due to their ECM-like structure involving high water retention potential, high porosity, and the possible presence of cell-adhesion ligands, hydrogels are often the material of choice in biofabrication^19^. However, they exhibit weak mechanical strength and stiffness, which challenge resolution and shape fidelity during fabrication^20^. In addition, nutrient and respiratory gas diffusion is limited to approximately 500 µm^21^. The aforementioned limitations pose a major hurdle, particularly for the biofabrication of muscle implants and meat structures, where nutrient delivery in several millimeter thick tissue as well as mechanical strength sufficient for implantation must be achieved.

**Fig. 2:**
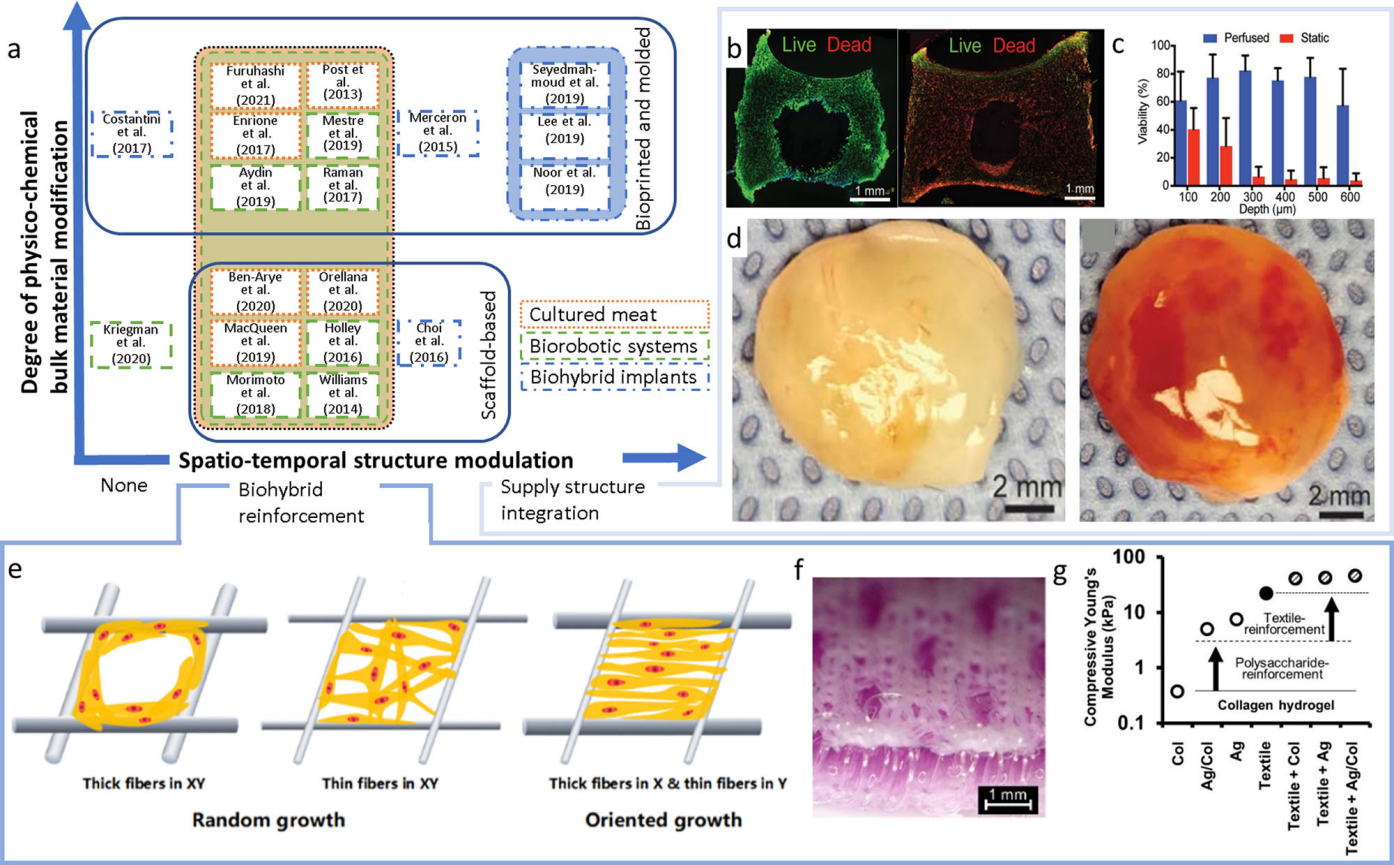
Categorization and examples of strategies for biofabrication of muscle tissue. Application-specific modi operandi for muscle tissue fabrication (**a**). According to the application, different degrees of physico-chemical bulk material modification and spatio-temporal structure modulation are applied for different examples found in the literature. The latter can be subdivided into biohybrid reinforcement (**e**–**g**), and supply structure integration (**b**–**d**). Live-Dead staining of actively perfused (**b**, left) vs. non-perfused (**b**, right) tissues as well as the measured cell viability as a function of the distance to the nutritional channel (**c**), exemplarily outline the importance of supply channel integration in thick tissues. The self-assembling capacity of vascular structures in bulk materials demonstrates the different potencies of compact (**d**, left) and highly porous scaffolds (**d**, right).(From ref. ^21^ Reprinted with permission from AAAS.) Biohybrid reinforcement was shown to strengthen cell alignment (**e**–**g**) and promote the mechanical properties of hydrogels (**f**, **g**)^23^ (difference in the morphological characteristics of a scaffold with different fiber diameters. by Xie et al (CC BY 4.0)). For instance, spacer fabric integration increased Young’s modulus of low concentrated collagen (Col) and alginate (Alg) hydrogels by several orders of magnitude (**g**)^22^ (Warp-knitted spacer fabric design and Morphological and mechanical analysis of warp-knitted spacer fabrics by Schäfer et al. (CC BY 4.0)).

To overcome these limitations and at the same time accommodate the application-specific demands (see chapter Application-specific muscle tissue fabrication), different strategies for muscle biofabrication evolved (Fig. 2a). These can be generally distinguished in bulk material modification (Table 1) as well as spatio-temporal modification strategies. The latter can be further classified in supply structure integration (Fig. 2b–d), such as the dedicated fabrication of vascular channels, and biohybrid reinforcement (Fig. 2e–g), where multi-material composites are generated to temporarily enhance the mechanical properties of the material^22^ or foster cell alignment^23^. Each strategy impacts different physico-chemical as well as biological properties of the biofabricated structure. Ultimately, the achievable properties need to be matched with the tissue-specific demands (Table 2) in order to select the most efficient strategies for the biofabrication of cultured meat, biorobotic systems, or biohybrid implants.

**Table 1:** Bulk material composition for the fabrication of cultured meat, biorobotic systems and biohybrid implants.

	Cultured meat	Biorobotic systems	Biohybrid implants
Cell types	Bovine myocytes from biopsies^13,35^ Bovine aortic smooth muscle cells (BSMC) and Bovine skeletal muscle microvascular endothelial cells (BEC)^11^ Mouse muscle myoblasts (C2C12)^12^	Mouse muscle myoblasts (C2C12)^16,37,127^ Neonatal rat myoblasts^2^ Primary rat cardiomyocytes^64,125,130^ Primary xenopus epidermis and cardiac progenitor cells^128^ Optogenetic mouse embryonic stem cell line ChR2H134R-HBG3 Hb9-GFP differentiated into motor neurons^55^	Mouse muscle myoblasts (C2C12)^21,24,57,58,66^ Human embryonic stem cells (hESCs) derived cardiomyocytes and endothelial cells^21^ Human primary muscle progenitor cells^36^ Mouse fibroblasts (BALB/3T3, NIH/3T3)^54,58,154^ Induced pluripotent stem cells (iPSCs)-derived cardiomyocytes and human endothelial cells or human umbilical vein endothelial cells^17^
Matrix materials and their concentration in the bulk	Collagen (type I): 2,4%^13^ Fibrin: 4%^13^ Matrigel: 20%^13^ Gelatin- Alginate scaffold: 7% (swollen), 90.5% (dry)^12^ Textured soy protein scaffold: 91%(dry)^11^	Collagen (type I): 2%^55^ Fibrin: 4%^37,127^, 2%^16^ Matrigel: 30%^37,127^, 2%^55^ Glycerol: 10%^16^ Gelatin: 3.5%^16^ Hyaluronic acid: 3%^16^	Collagen (type I): 1.2-2.4%^21^ Fibrin: 4%^21^, 2%^36,66^, 2.5%^54^ Decellularized muscle extra cellular matrix: 2.5%^17^, 1%^24^ Glycerol: 10%^36,66^ Gelatin: 3.5%^36,54,66^ Hyaluronic acid: 3%^36,54,66^ Alginate: 4%^21^, 6-8%^57^, 4%^58^ Polyethylene glycol (PEG)- Fibrin: 0.8%^58^ Methacrylated Gelatin (GelMa): 10%^57^ Methacrylated hyaluronic acid: 1.5%^21^
Crosslinking mechanisms	Biological (enzymatic), Physical (thermal)	Biological (enzymatic), Physical (thermal)	Biological (enzymatic), Chemical (UV-light assisted covalent crosslinking) Physical (thermal, ionic)
Support materials for fabrication		Polydimethylsiloxan (PDMS)^2^	Gelatin microparticles support bath^21^ Sodium alginate microparticles (0,32 %) and xanthan gum (0.25 %) supplemented support bath^17^ Pluronic F-127^66^
Support materials for tissue conditioning	PDMS pillars^13^ Agarose hub^35^ Textured soy protein^11^ Gelatin- Alginate scaffold^12^	Polyethylene glycol -diacrylate (PEGDA) –skeleton^37,127^ PDMS pillars and substrates^2,64,125,130^	Oxygen releasing particles^57^ Polycaprolacton (PCL) anchors^24,36^ Polyurethane (PU) grids^54^ PCL grids^54^

**Table 2:** Application specific properties and requirements of biofabricated muscle tissue.

Application specific requirements	Cultured meat	Biorobotic systems	Biohybrid implants	Implementation methods
*General requirements*				
Cost/ Resource efficiency	**+++**	+	o	Structuring methods Bulk material modifications
3D resolution and shape fidelity	o	+	+++	Structuring methods Mechanical reinforcement Crosslinking strategies
Size	++	o	++	Mechanical reinforcement Porosity and vascular channel integration
Individualization	+	o	+++	Autologous cell sourcing Native tissue imitation
*Biological requirements*				
Vascularization and innervation potential	o	+	+++	Cell sourcing Porosity and vascular channel integration
Prolonged cell viability	o	+	**+++**	Cell adhesion and migration Porosity and vascular channel integration
Controlled force output	o	**+++**	+	Tissue innervation Cell adhesion and migration Mechanical stimulation for conditioning Directed force generation Porosity and vascular channel integration
Metabolic activity	+	+	+++	Bulk material modifications Mechanical stimulation for conditioning
Short term biodegradation	+++	o	o	Bulk material modifications Porosity

The properties are evaluated from very significant (+++) to indifferent (o) for each application. The dominating properties for each type of application are highlighted in bold.

### Physico-chemical composition of the bulk material

For the fabrication of muscle tissue different cell types (e.g. myoblasts), cell media, matrix materials (e.g. hydrogels), and supporting materials need to be joined in a coordinated way. While this sub-chapter focuses on the general bulk material characteristics, the following sub-chapter emphasizes the biofabrication strategies that describe the spatio-temporal arrangement of the individual components. The following paragraphs outline how selection and composition of the bulk material differ for the three fields of applications: cultured meat, biorobotic systems, and biohybrid implants (Table 1).

While muscle-specific cells are the key component in muscle biofabrication, the types and sources might differ according to the application as either cultured meat, biorobotic system, or a muscle implant. Due to its high availability and well-studied nature, the immortalized mouse myoblast cell line C2C12 has already been used in all three applications^12,16,24^. To improve the mechanical properties, texture or functionality myoblasts were observed to be frequently combined with fibroblasts in muscle implants and cultured meat^11,17,25,26^.

The combination of different cell lines not only increases the difficulty of cell culture due to the cells’ individual demands for cell culture media^27,28^, it also can improve the outcome by recreating the native composition of the muscle^9^. For instance, co-culture with fibroblasts has been shown to improve cell maturation^29^. While co-culture with motor neurons was shown to improve the innervation rate and functionality^30^. In addition, combining muscle cells with endothelial cells and fibroblasts can improve angiogenesis, leading to improved graft efficiency when used as muscle implants^27,31^ and thereby also increasing the muscle tissue functionality^31^. The co-culture with fat cells was shown to support the taste of the muscle^32^.

Though cell lines would be conducive to achieving the goals of an affordable, animal-free product^33^, for cultured meat the cell line of choice still needs to be developed. Apart from the quail originated QM7^34^, currently applied cell lines do not represent typically eaten animals, such as pig, cattle or chicken. For this reason, primary cells from bovine biopsies are routinely applied^13,35^, contradicting the original goal of an animal-free product. Understandably, human cells are used for muscle implants only^17,21,36^. Induced pluripotent stem cells differentiated into cardiomyocytes (CMs) or endothelial cells could be used to achieve the goal of patient-specific implants^17^.

Interestingly, genetically engineered cells are a popular choice in biorobotic systems. Integration of environmental triggers broadens their field of application compared to conventional technical actuators. Mostly cells with light-sensitive cation channels were used which contract when triggered by a light impulse. Those cells have no need for invasive stimulation by electrodes, enabling further miniaturization and non-restricted movement^37^. Additional modification methods could enable biorobots with enhanced contractile force^38^, chemotactically driven robots for in vivo drug delivery^39^, or biodegradable robotic systems with integrated microplastic-degrading bacteria that might clear water from microplastics in the future^40^.

Cell media provide the cells with nutrients, the required osmolarity and pH. The animal serum is considered the gold standard for the supply of growth factors in cell culture media. In this regard, its application is crucial for cell expansion, cell adhesion, or differentiation. For example, usually approx. 10 % fetal bovine calf serum or 2 % horse serum are routinely supplemented for cell expansion or differentiation, respectively^41^. Though essential for cell culture, the usage of animal-derived serum is critically discussed both in society and industry^42^. In particular, its mammal-derived origin and extraction, which are associated with animal harm, are the center of controversial debates^43^. In addition, the high cost is a major hurdle for the price targets of cultured meat companies^42^. Moreover, its batch-to-batch variations represent an issue in quality management^44,45^. Finally, when applied in the field of regenerative medicine it bares the risk of inducing animal-borne diseases or causing immunogenic reactions in humans^41,46^. For the above reasons, scientists have long been looking for alternative medium supplements, such as synthetic serum. Due to its wide range of applications the replacement of animal serum is of high interest in the field of bioartificial muscle engineering^42^. For example, different serum-free media have been successfully applied for maturation of C2C12 muscle cells^41,47^. With the goal of providing biological meat without animal suffering, the pressure to avoid animal serum is particularly strong for cultured meat applications. For co-culture of cell types, e.g. myoblasts and endothelial cells, Dulbecco’s Modified Eagle’s Medium (DMEM) or a combination with a different medium can be used^27,31^.

For the biofabrication of muscle tissue, different matrix materials, such as hydrogels (for bioprinting and molding methods)^16,17,19,48^ or porous scaffolds and thin films (for cell seeding)^11,49^, are applied. In order to generate a functional tissue, they have to fulfill specific biological and mechanical requirements. Some of these properties are generic for tissue engineering^50^, e.g. the presence of tripeptide RGD-binding motifs to support cell adhesion and migration. Other properties are muscle tissue specific, such as the matrix elasticity, which is known to influence cell fate^51,52^. Considering this, the mechanical properties of hydrogels and scaffolds applied in muscle tissue fabrication should be similar to their natural counterpart, approximately 12 kPa^53^, to support cell differentiation and aid the formation of myotubes^6^.

In order to match the described requirements and enhance the biological acceptance of the hydrogels’ bulk material, different physico-chemical modulation strategies are applied (Table 1). In muscle fabrication, this is mostly achieved by modifying the polymer type and concentration, applying multi-material hydrogel formulations (hydrogel blending)^13,16,54^, or adding support structures (e.g. polydimethylsiloxane (PDMS) pillars)^16^.

Collagen (type I), fibrin, and Matrigel are the most prominently applied matrix materials and can be found in all three fields of application due to their excellent biological properties^13,21,55,56^. Interestingly, in muscle implants as well as meat blending of the protein-based matrix materials with polysaccharides, such as agarose^12,35^ and alginate^57,58^, can be observed. The reasoning for this could be found in previous studies, where polysaccharide blending was shown to be a simple, yet promising, tool to modulate the mechanical properties as well as the microstructure of the bulk matrix^59–61^. The group of Levenberg recently published a very innovative edible scaffold matrix formulation including textured soy protein specifically designed for meat fabrication^11^. Besides the type, we found that the applied concentration of the matrix polymer also strongly varied between the different applications. While matrices designed for muscle implants exhibited rather low concentrations (1.2–10%)^21,36,57,58^, for meat fabrication matrices with high polymer content (20.6-91.0 %) and only minor moisture (9 %) were used^11–13^.

Furthermore, slight differences in the applied crosslinking mechanisms can be found. All three fields of applications involve physical (thermal^17,35,55^ or ionic^12,57,58^) as well as enzymatic crosslinking (thrombin^13,16,36,54^). Interestingly, only for muscle implant fabrication we found the application of ultraviolet (UV)-crosslinking methods^58^, although the potentially toxic and carcinogenic side effects of UV-light and photoinitiator exposure can be considered most critical in this field of application^62,63^.

In all three fields of application, support materials are of the highest importance. They either aid the manufacturing process, serve as a guidance to promote cell alignment, or facilitate the fastening and subsequent conditioning of the muscle^13,16,36^. Mostly, the applied support materials (e.g., polyethylene glycol diacrylate (PEGDA), PDMS, polycaprolactone (PCL)) exhibit a higher stiffness, yet a comparably high biocompatibility, in contrast to the employed cell-laden hydrogels^37,54,64^. Obviously, in cultured meat applications edible support materials, such as soy proteins, are used as mechanically supporting and rheology-modifying filler^11,12,49,65^. In muscle implants and a few biorobotic applications, where complex geometries and vascular channel integration are aimed for refs. ^13,17^, the additional employment of temporary sacrificial materials^36,66^ and support baths with integrated microparticles, such as xanthan gum^17^ or gelatin^21^, can be observed. Interestingly, one approach even integrated oxygen-releasing particles to improve cell viability^57^.

The natural ECM exhibits biochemical and topographical signals, which influence cell behavior like cell viability, morphology, proliferation, and differentiation^9,67^. The physico-chemical properties of various scaffold materials can also be used to aid muscle tissue formation. For example, cues for differentiation could be recreated, by including specific catalysts in the matrix material, such as graphene. In recently published studies graphene was shown to induce spontaneous myogenic differentiation without additional chemical cues by enhancing the adsorption of fibronectin and albumin and upregulating intercellular signaling^67–69^.

For highly biofunctional tissues it is important that a number of cells remain in an undifferentiated quiescent stem cell state, as those cells have the ability to repair and repopulate a tissue to enhance the therapeutic efficacy of muscle implants^70^. In order to modulate cell cycle progression, regulate the cell−matrix interaction, and mediate the cytoskeleton remodeling processes, creating stem cell niches plays a vital role^71^. The stiffness of the niches’ growth environment, as well as the presence of specific neighboring cells, molecules and growth factors are essential in this context^70,71^.

### Spatio-temporal modification

While the previous sub-chapter outlined the effect of matrix material composition, this chapter gives an overview on different spatio-temporal modification methods that are applied to these. Spatio-temporal modifications result in hierarchically structured material properties that are modulated over time. In general, two categories are distinguished: strategies for nutrient supply channel integration and strategies for biohybrid reinforcement. The former benefits cell viability by improving nutrient supply. The latter enables modulation of the structure’s elastic modulus, supporting shape fidelity, cell maturation, and cell alignment necessary for myotube formation. In sum, both strategies have a huge impact on long-term functionality of the tissue and differ for the three applications of interest: cultured meat, biorobotic systems, and biohybrid implants.

In biofabricated thick muscle flaps, limited nutrient supply and metabolic waste product removal can be observed in tissues exceeding 100 µm for static and 600 µm for perfused culture, resulting in deteriorated viability and necrotic core formation^21^ (Fig. 2b, c). In native tissue, this issue is addressed by the presence of a dense vasculature network. In bioartificial muscles this approach can be mimicked by integration of nutrient supply channels. Depending on the biofabrication approach and required lifespan, different strategies for nutrient supply channel integration can be applied^72^.

Our investigations reveal that the way nutrients are provided strongly differs for the three fields of application. While thin tissues, most frequently found in biorobotic systems^16,56,73^ and thin cell-seeded scaffolds for cultured meat^11,12,35^, can be supplied via diffusion, tissues exceeding a thickness of 500 µm require active nutrient supply^21^. Depending on the application, the latter might be required for short term (up to 4 weeks) or long term (> 4 weeks) (Table 2). For instance, while short-term nutrient supply is sufficient for cultured meat^11,74^, long term functionality needs to be provided for skeletal muscle implants. The applied strategies for nutrient supply channel integration reflect this observation.

Thick muscle tissue intended for cultured meat applications can be fabricated applying molding techniques in combination with channel forming materials or porogens in order to yield highly porous open lumen scaffolds. While the channel forming materials vary, the molding process is quite similar in most studies found in the literature. First the channel forming component, e.g. dissolvable phosphate glass fibers^25^, carbohydrates^75^, or sacrificial hydrogels, such as Pluronic F-127^36,76^, are brought in the desired 3D-shape. Most frequently, 3D-printing or molding methods are the method of choice for this purpose. Subsequently, the channel structures are placed in the mold before the cell-laden hydrogel is casted into it^21,76^. Finally, the void-forming components are removed either mechanically (e.g. pulling it) or physico-chemically (e.g. by dissolving it), resulting in a bulk material with integrated open lumen structures^72,75,77^.

For muscle implants that require long-term nutrient supply, more sophisticated methods need to be applied. In order to generate vessel-like structures that (i) ensure long-term stability, (ii) prevent occlusion of the nutrient channel, and (iii) enable biological compliance with the host tissue, organized channels rather than open lumen pores are required and their interfacing walls should be lined with endothelial cells. In this context, 3D-bioprinting technology is mostly applied. Here, nutrient supply structures comprising sacrificial hydrogels are printed in parallel with muscle cell containing matrix materials. Following the printing process, the sacrificial materials can be dissolved to generate oriented and perfusable open lumen vessel-like structures^78^. Other approaches employ co-axial bioprinting methods to generate multi-layered material strands comprising one or more cell-laden matrix materials and a core made of a sacrificial material, air or cell culture media^20,79^. In both attempts, endothelialization of the open lumen structure can be achieved, by including endothelial cells and human umbilical vein smooth muscle cells within the sacrificial material or by subsequently flushing the hollow structures with those^17,79^. In sum, long-term stable vascular structures with high biofunctionality can be readily fabricated^77^.

Besides 3D-bioprinting, self-assembly methods are an innovative approach to create vessel-like conduits for long-term nutrient supply. In this context, two mechanisms are distinguished: intrinsic and extrinsic vascularization. Both can be controlled by spatio-temporal release of angiogenic factors like vascular endothelial growth factor (VEGF)^77,80^. In the former, pre-vascularization of tissues occurs, e.g. by co-culturing endothelial cells and stem cells in the bulk material of the biofabricated structure^81,82^. Extrinsic vascularization relies on implantation of a fabricated porous structure^21^. Open lumen structures and micropores strongly support extrinsic vascularization. Both act as guidance and provide space for developing vascular channels that sprout from the natural tissue into the fabricated bulk^25^. High levels of microporosity are required for this step, which can be implemented by embedding porogens or sacrificial microparticles in the hydrogel precursor solution. After structuring the bulk material, those porogens can be dissolved, leaving micropores for vascularization^21,83^. Pore sizes ranging from 160–270 µm were shown to assist vascularization, e.g. in Polyethylene glycol (PEG)-scaffolds^5,84^. Aligned microfibrils fabricated for example by shear-extrusion or electro writing could also be included during biofabrication of muscle tissue in the future, as it was shown that they can aid the formation and patterning of vasculature from iPSCs^85,86^. However, both the extrinsic and intrinsic approach require at least 1-2 weeks of pre-cultivation before the vascular network is formed and functional, resulting in lacking nutrient supply during this period^87,88^. To prevent necrotic core formation, the described self-assembly methods are mostly applied for thin tissues only^21^ (Fig. 2d) or in combination with additional spatio-temporal bulk material modification methods to bridge the gap in nutrient supply.

Biohybrid reinforcement describes the support of mechanically weak hydrogels or cell suspensions by combining these with more rigid materials, which may degrade over time. The support structures, which mostly reflect anisotropic orientation, offer both mechanical and surface cues for cells in distinct regions of the biofabricated tissue. In this context, reinforcement was shown to impact not only the mechanical properties, but also 3D shape fidelity, tissue conditioning as well as maturation, and ultimately the power transmission potential of biofabricated muscle^14,16,66^. In particular the latter is key in implants and biorobotic applications.

A high variety of rigid biocompatible materials from the natural origin such as collagen, gelatin, soy protein, silk fibers and synthetic polymers like polylactic acid (PLA), polyglycolic acid (PGA) and PCL are applied to reinforce hydrogels and cell suspensions^11,21,54,89–94^. For muscle tissue fabrication elastic materials, such as PDMS and Polyurethane (PU), are of special interest. They possess high elasticity and enable repeated stretch cycles without aging or showing signs of deteriorated mechanics. For this reason, they have been employed successfully as muscle tissue reinforcement in the past^95,96^. For biohybrid implants usually materials that are biodegradable, such as PLA, PGA, or PCL^89^ are favored to enable restructuring processes of the tissue implant from the hosts’ cells system to achieve a fully integrated functional implant^97^. Finally, muscle tissue intended for meat applications demands edible reinforcing materials. Commonly applied candidates comprise materials that are generally regarded as safe (*gras*) like starch, alginate, soy-proteins, or gelatin^12,49,98,99^.

Besides biological and chemical properties, myoblast alignment and an elastic modulus that is similar to the natural tissue are crucial for muscle tissue maturation and in particular for myotube formation^6,52,100^. The usually low elastic modulus of hydrogels, compared to natural muscle with about 12 kPa^100,101^, results in a reduced functionality^14,66^. By including scaffolds as biohybrid reinforcement, elastic modulus and cell alignment can be altered (Fig. 2e–g)^22,23^. Elasticity and cell alignment of the biofabricated composite are influenced by the material properties, the quantity, the orientation and the design of the rigid reinforcement structures^23^ (Fig. 2e).

Pre-fabrication of elastic scaffolds that are subsequently seeded with cells is a commonly applied reinforcement method for muscle tissue generation^11,89,102^. To fabricate comparably thin cell sheets, which are often found in biorobotic systems^64^ and cultured meat^94^, flat substrates such as films with unidirectional groves, electrospun fiber substrates, or knitted textiles can be applied (Fig. 2f, g)^22,103^. Besides mechanical reinforcement, thin sheets foster cell alignment via contact guidance^104,105^. Thicker muscle tissues can be generated by applying anisotropic scaffolds, produced by directed freeze drying^11^ or fused filament fabrication^106^. These methods enable controlled modulation of the elastic modulus as well as the implementation of cell alignment features. For instance, freeze-drying techniques were successfully employed to manufacture anisotropic sponges, which provided aligned and interconnected pores to stimulate myofiber development^11,12^. In contrast, scaffolds with isotropic pore orientation result in randomly directed cell strands^107^. In general, the strategy of pre-fabricating scaffolds offers a comparably high level of freedom with respect to the applicable materials and processing methods. Since the scaffolds can be manufactured in advance, non-cytocompatible production methods, such as toxic crosslinkers, non-physiological temperatures, or prolonged production times can be accepted. Prominent examples are the temperature and pressure critical freeze drying process to create porous scaffolds^11,12^, or the high mechanical stress exerting and potentially lubricant-containing textile manufacturing^22^. However, the comparably low spatial resolution that can be achieved in the subsequent cell seeding step is a critical drawback of this approach. To overcome this shortcoming, recently hybrid 3D-bioprinting methods have gained attention. In contrast to the previously described cell seeding approach, the reinforcing components and the cell-laden matrix material are deposited not subsequently, but in parallel. For instance, thermoplastic polymers like PCL and PU have been printed together with mouse myoblast-laden fibrin-composite bioinks. Thus, bioink and supporting material can be joined to from muscle tissue with high spatial orientation. The printed polymer strands provided both reinforcement as well as surface-guiding cues resulting in a defined orientation of the deposited cells promoting myotube formation^54,66^.

Ultimately, the formation of aligned myotubes can be fostered by mechanical stimulation. Interestingly, so far mostly isometric instead of cyclic strain is applied in this context, while the benefits of the latter are controversially discussed^108,109^. Isometric strain is achieved either by printing or molding the cell-laden matrix around rigid anchor points. Subsequently, crosslinking as well as cell-driven remodeling of the matrix result in a time-dependent shrinkage of the tissue. The shrinkage-induced isometric strain is a simple, yet powerful method to assist cell alignment and myotube formation in the resulting tissues^13,56^.

## Application-specific muscle tissue fabrication

Biofabricated muscle tissue can be applied for versatile purposes, e.g. as cultured meat, in biorobotic systems, or as biohybrid muscle implant. For each of the three applications, individual tissue specifications dominate. To accommodate these, the biofabrication process has to be tailored accordingly. The following sections provide an overview on the applications’ specific requirements and their implementation methods (Table 2). Recent examples and success stories for each respective area of application are highlighted.

### Cultured meat

Cultured meat is emerging to fulfill the growing world population’s desire for animal- and environment-friendly meat^46^. Several companies are actively researching the production of cultured meat, but at this point no product is commercially available at a large scale^110^. It consists of expanded differentiated cells in combination with other scaffold materials for nutrition. Apart from consumer acceptance, the product predominantly has to be cost and resource efficient to succeed in the food industry^5^. For this reason, muscle cell lines, which exhibit a vast proliferation potential, are routinely applied and expanded for mass production in bioreactor systems. The perfusion bioreactor is the most commonly used reactor type, yet the most resource efficient in terms of space and culture media is the hollow fiber reactor^74,111^.

To further expand the mass of cultured meat, additional natural edible materials are added^12,65,112^. There are two possible product types of cultured meat: a highly structured cut meat or a low textured minced meat. For the fabrication of low structured products, cell seeding of preformed scaffolds^11,12^ or molding methods^13^ are mostly applied. To achieve a higher order of cellular structure, more advanced but so far less scalable biofabrication methods, such as 3D-bioprinting, are in favor. For example, to create cut-meat-like products, different cell types, such as muscle and fat cells, have to be placed with a high spatial resolution to achieve the best look and taste^32^. Furthermore, a dedicated maturation step needs to be added, in order to achieve myofiber formation and orientation and ultimately create a textured meat product^6,23,74^.

The key requirement for the application of muscle tissue in the context of cultured meat is the scalability of the production process and customer acceptance to be financially profitable^113^. This demand has a huge impact on the biofabrication process. Most studies focus on yielding high quantities rather than the functionality of the muscle, which is not necessarily required in this field of application. This chapter highlights current manufacturing trends in this rapidly evolving field of research.

**Fig. 3:**
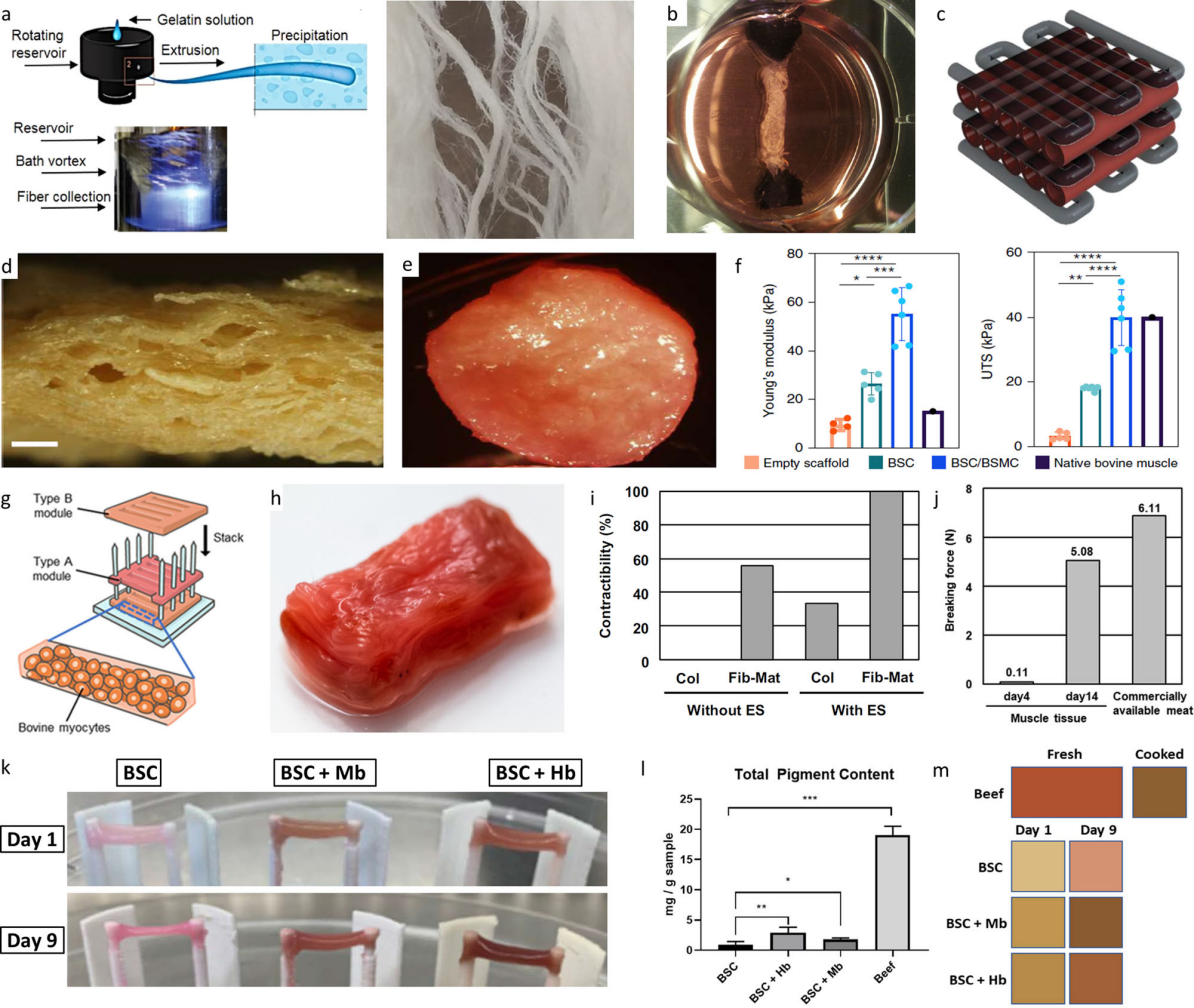
Recent trends in cultured meat fabrication. Fibrous anisotropic gelatin scaffolds could be produced by immersion rotary jet spinning (**a**)^94^ (fibrous gelatin production by immersion rotary jet spinning (iRJS) by MacQueen et al. (CC BY 4.0)). Picture of a muscle strip with anchoring system which was further developed for the first burger from cultured meat (**b**)^108^ (Reprinted from Principles of Tissue Engineering, Fourth Edition, M. Post, C. van der Weele, Principles of Tissue Engineering for Food, Pages 1647–1662, Copyright (2014), with permission from Elsevier). Schematic depiction of a possible cultured meat scaffold design (**c**)^100^ (Reprinted with permission from Springer Science Business Media, LLC, part of Springer Nature: Food Engineering Reviews, Cultured Meat: Meat Industry Hand in Hand with Biomedical Production Methods, Zidarič et al., Copyright (2020)). Textured soy protein scaffold (**d**) seeded with bovine satellite cells (BSC) and bovine aortic smooth muscle cells (BSMC) co-culture (**e**). Comparison of Young’s modulus and ultimate tensile strength (UTS) of the various seeded textured soy scaffold types of native bovine muscle from the literature (**f**)^11^.(Reprinted with permission from Springer Nature Limited: Nature Food, Textured soy protein scaffolds enable the generation of three-dimensional bovine skeletal muscle tissue for cell-based meat, Ben-Arye et al, Copyright (2020)) Construction process of a thick bovine muscle tissue (**g**) and image of the resulting product colored using red food dye (**h**). The rate of fiber-shaped bovine muscle tissues capable of contracting in response to applied electrical stimulation (ES), formed within the collagen (Col) or Fibrin-Matrigel (Fib-Mat) based tissue cultures (**i**) and mechanical characterization of the produced tissue (**j**)^13^(Construction process of millimetre-thick bovine muscle tissue, Morphological and functional analysis of bovine muscle tissue, Morphological analysis of the millimetre-thick bovine muscle tissue and Food feature analysis of the large bovine muscle tissue by Furuhashi et al (CC BY 4.0)). Representative images of cultured meat strips of bovine muscle satellite cells (BSCs) grown in the presence of hemoglobin (Hb) or myoglobin (Mb) in the cell culture media for up to nine days in a fibrin hydrogel (**k**). Spectroscopic quantification of total pigment content (**l**) and average tissue coloration (m) of homogenized cultured meat strips after incubation in heme-protein-containing media in comparison to beef^121^. (properties of skeletal muscle tissue formation and Pigment content and tissue coloration by Simsa et al. (CC BY 4.0)).

Initial proof of concept studies on cultured meat employed animal-derived scaffolds, simple and thin geometries as well as low levels of cellular organization and texture. In this context, perfusion or hollow fiber bioreactors, as well as cell expansion in cell aggregates or on microcarriers were most often applied to produce large amounts of muscle cells^74,114^, which could be further supplemented with proteins and processed into the desired shape^15^. Adding to or replacing muscle cells by less expensive, alternative protein sources, was shown to be an effective way in reducing costs in order to offer cultured meat at a competitive price. For this purpose animal-derived polymers like collagen or gelatin (Fig. 3a), which offer excellent cell growth conditions, high biodegradability, and weak antigenicity, were frequently reported^11,35,94,115^. Recently, inexpensive and animal-free polymers are gaining attention as a promising alternative to the aforementioned additives^11,112,116^. However, non-ECM-sourced hydrogels most often lack biofunctionality, e.g. cell-adhesion ligands. In this context, transgenic plants, which can be tweaked to produce collagen-like materials^117^, could be a key for future developments^5^.

Following the early proof of concept studies, more advanced methods were employed to increase complexity and resemble the anisotropic features of native meat. For example, the fabrication of the first lab-grown meat burger by Post and his team in 2013, which marked a milestone in cultured meat development, isometric strain conditioning was employed to support cell alignment and tissue maturation^35^ (Fig. 3b). More recently, ambitions to recreate sliced meat resembling products increase and growing efforts for fostering tissue maturation and cell alignment by post-fabrication conditioning can be observed. For instance, Ben-Arye and co-workers applied a freeze-drying process to create a scaffold with oriented and interconnected pores. In combination with a soy protein, thin textured meat slices with good biological properties could be generated^11^. Other groups focused on micropatterned or grooved surfaces, e.g. gelatin–alginate–agarose–gylcerol-composite films or gelatin fibers sheets, to promote cell alignment^49,94^. Several other edible materials, such as cellulose, fungal chitosan and pectin would be favorable alternatives but have not yet been applied for the production of structured meat^112,116,118^.

To be accepted as food from the consumers, cultured meat needs to achieve the look and taste of its native counterpart. Cell seeded scaffolds dominate current meat fabrication strategies, but are yet to be analyzed regarding their taste. Most scaffolds are neutral in taste, for example gelatin or starch, and could be used for all kinds of cultured meat products, while seaweed-derived alginate is a commonly applied alternative in seafood^118^. The taste of the few cultured meat products already degusted were described as meat-like^11,108^, though considered not to be the same as real cut meat. Important differences are the missing fat content^119^ and various proteins^120^. Recent research underscores the increasing importance of this aspect and points to exciting approaches to solving it. For instance, Simsa et al. elucidate the role of heme proteins, e.g. myoglobin and hemoglobin, in tailoring the bloody-metallic taste typically experienced when eating cut meat^121^. In addition, the heme proteins were shown to improve the visual appearance of the cultured meat products in contrast to the light pink color of untreated cell cultures^121^ (Fig. 3k–m). Other approaches apply food coloring agents, such as beet juice, to improve the visual acceptance and achieve a raw meat mimicking look^11,13^ (Fig. 3e, h).

With increasing demand for mimicking its native counterpart, the texture or mouthfeel of cultured meat was also brought into focus recently. Both aspects are directly influenced by the manufacturing process and the subsequent conditioning procedure. For instance, using edible porous soy scaffolds in combination with muscle and connective tissue-derived cells (bovine satellite cells (BSC), bovine aortic smooth muscle cells (BSMC) and bovine skeletal muscle microvascular endothelial cells (BEC)), a meat product that matched Young’s modulus and the ultimate tensile strength of native bovine muscle (Fig. 3f) was generated^11^. Besides the mechanical properties, the rheological features can also be used to analyze the texture and quality of cultured meat products^94^. For instance, MacQueen and co-workers created a muscle cell-seeded gelatin scaffold, which recapitulated some of the structural and mechanical features measured in meat products. Here, an immersion rotary jet spinning approach together with a chemical and enzymatic crosslinking strategy were applied for meat matrix fiber fabrication^94^ (Fig. 3a). Lately, multi-modal approaches involving isometric strain (anchoring systems), spatio-temporal matrix modification (inclusion of channels for structuring and nutrition supply) as well as electrical stimulation are gaining particular attention to match the increasing demands in cultured meat fabrication^13^. Following this strategy, Furuhashi and co-workers generated meat structures that exhibited similar mechanical properties compared to native muscle tissue^13^ (Fig. 3g–j).

Interestingly, animal serum is still frequently used, despite the growing desire to produce cultured meat without animal-derived products. Moreover, efforts to find cost-efficient solutions are at their greatest in this area^11,13,98^. The use of synthetic serum would therefore make an important contribution in many respects and therefore offers a promising future.

As discussed earlier (Chapter Spatio-temporal modification), due to its limited lifespan short-term nutrition supply is sufficient for cultured meat. Recent studies either realize this by applying thin, diffusion enabling sheets up to 1500 µm^11,13,35,49,94^, or by taking advantage of low oxygen and low nutrient consuming cell types, e.g. fish cells^122^. Interestingly, only recently first strategies towards the fabrication of perfusable, thick meat structures, were employed^33,100^ (Fig. 3c).

### Biorobotic systems

Biorobotic systems are biologically driven actuators using hybrid constructs comprising force-generating muscle cells that interact with an underlying substrate or a surrounding matrix material to generate motion.

In contrast to cultured meat, yielding a high level of biomechanical functionality and a high force generation capacity are of utmost importance for these systems. Depending on the field of use, different muscle cell types can be employed. For instance, muscle cells derived from insects are well suited for ex-vivo applications. They are characterized by a high force generation capacity, prolonged actuator lifetime (up to 90 days), and can be operated at atmospheric conditions with only minimal nutritional supply^73,123,124^. In turn, primary human muscle cells are advantageous in that they can be employed within the body without risking immunological side effects. However, compared to insect muscle these exhibits a shorter actuator lifetime (< 20 days)^124^. Despite species-related differences, the selected cell types were shown to impact the performance of biorobotic systems too. For example, the generated force of cardiomyocytes (approximately 10 µN/cell) is about ten folds higher than the power of skeletal muscle-driven actuators (approximately 1 µN/cell)^124^.

The high miniaturization potential is one of the strongest edges of biorobotic systems compared to technical actuators, which cannot be scaled down in size as much^125^. Due to their comparable small size and weight, biorobotic systems exhibit an excellent power-to-weight ratio, which is approximately 1–3 orders of magnitude higher than for instance pneumatic systems, or electroactive polymers^124,126^. Furthermore, the applied muscle cells can be genetically modified to react on versatile environmental triggers, such as light or chemical cues, in real time^37,124^. Last but not least, biorobotic systems are superior to technical actuators in that they exhibit self-healing properties which make them resilient to mechanically-induced damage^127^. The high miniaturization potential, their wireless power supply (powered by dissolvable nutrients), and their versatile triggers renders biorobotic systems ideal for in vivo applications, for instance for future drug delivery, or plaque removal tasks^124,128^.

So far, biorobots comprise rather thin muscle tissue patches (approx. 500 µm) with simple shapes^64,73,123,127,129,130^. For this reason, biomaterials and biofabrication processes that do not need to yield high shape fidelity or hierarchical designs are predominantly applied.

**Fig. 4:**
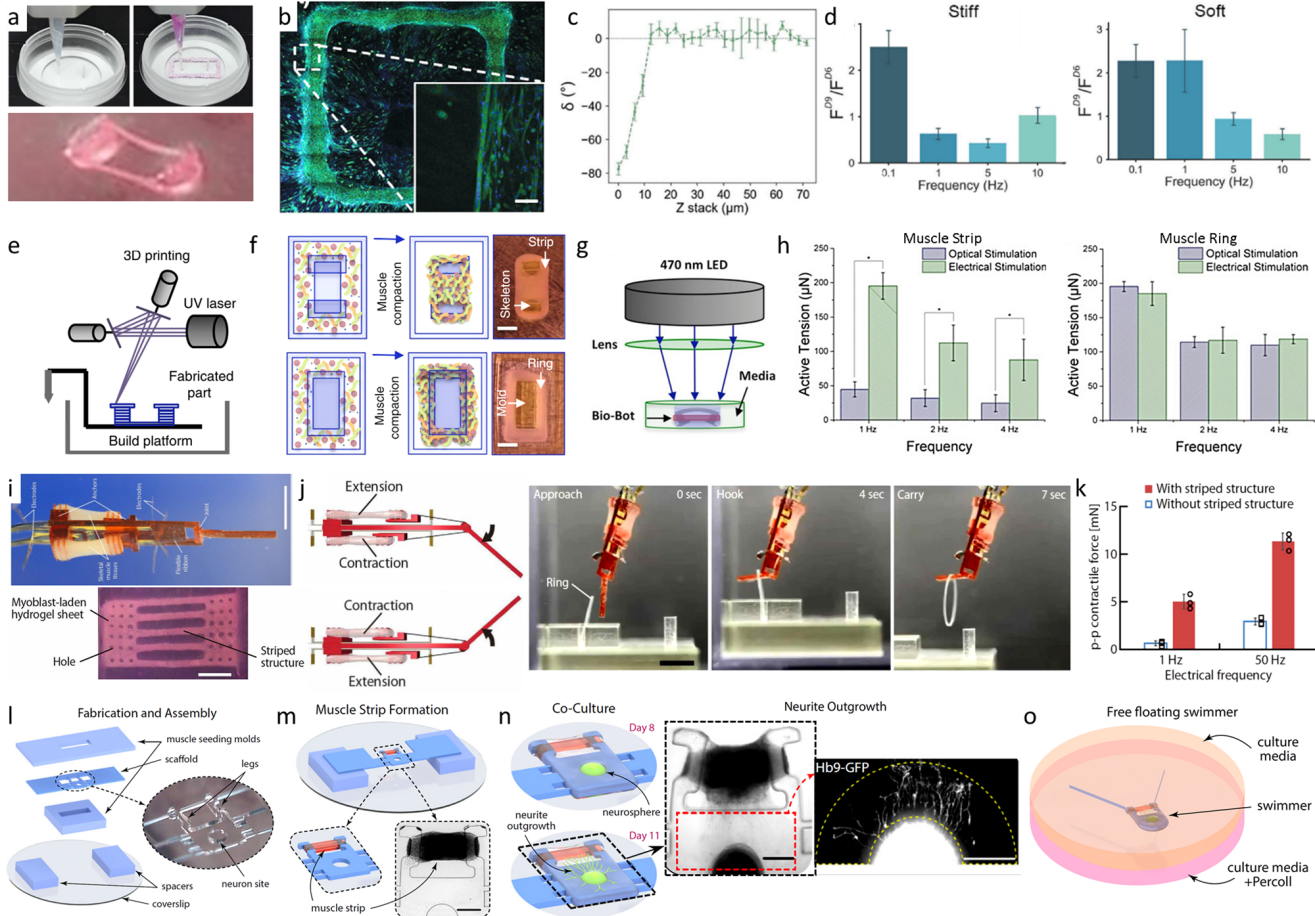
Examples of bioprinted and molded biorobotic systems. Extrusion printing of muscle cell laden bioink and PDMS pillars for cell alignment (**a**). Microscopic image of the print (**b**). Myotube alignment in the printing direction and in parallel to the passive force from the pillars (**c**). Fold increment of the achieved force of the cells before (F^D6^) and after 4 days of stimulation (F^D9^) with differing stiffness of the pillars (**d**)^16^ (© 2018 WILEY-VCH Verlag GmbH & Co. KGaA, Weinheim) Schematic manufacturing process of muscle strips and rings with stereolithographic 3D printed molds (**e**), injection of the bioink and following muscle compaction to achieve muscle strips and rings for bioreactors (**f**). Actuation of the genetic modified muscle cells via optical stimulation (**g**) and electrical stimulation (**h**). Active tension changes with the stimulation method and the muscle shape **h**)^37^. Biohybrid robot powered by an antagonistic pair of skeletal muscle tissues (**i**). Example movement of the biorobot grabbing a ring (**j**). Contractile force is increased with a sufficient maturation and therefore striped structure of the muscle cells (**k**) (from ref. ^2^. Reprinted with permission from AAAS.) Biofabrication of a free floating biorobotic swimmer driven by on-board neuromuscular unit (o) with the steps of fabrication and assembly (**l**), muscle strip formation (**m**), co-culture of muscle cells and the neurosphere in a continuous ECM-gel (**n**) (from ref. ^55^ Biofabrication timeline and free swimming driven by neuromuscular units by Aydin et al. (CC BY NC ND 4.0)).

In addition, its limited dimension simplifies nutrient and oxygen supply of the involved cells, since these can be supplied by diffusion. For fabrication of biorobotic systems mostly molding, bioprinting or cell seeding are applied. For molding or bioprinting of muscle tissue, ECM derived hydrogels, such as collagen and Matrigel, are routinely applied at low polymer concentrations as these formulations offer excellent cell growth conditions and thus high cell densities^2,16,37,55,131^. In some cases, these are additionally blended with e.g. fibrin or Methacrylated Gelatin (GelMa) to improve the mechanical properties of the bulk material^127,129,131^. The matrix material is usually casted or printed onto strips or annular molds creating multi-material constructs^37,56^. For example, Morimoto *et al*. used a striped structure in the mold as reinforcement to aid both cell alignment and nutrition^2^. Recently, more complex structures were fabricated using 3D-bioprinting technology (Fig. 4a–h)^16,37^.

Following the fabrication process, the generated structures need to be transformed into functional, contractile biorobots. In literature, this is most often achieved by promoting cell alignment or exerting isometric strain^2,16,37,129^. The maximal force that can be exercised by the biorobotic system depends on multiple factors, such as the mechanical properties of the substrates or the muscle fixation posts^16^ (Fig. 4d), the muscle stimulation and conditioning method^37^ (Fig. 4g, h), and the level of tissue maturation^2^ (Fig. 4k). Additionally, co-culture with neurons enables new possibilities for the design of biorobotic systems^55^ (Fig. 4l–o).

**Fig. 5:**
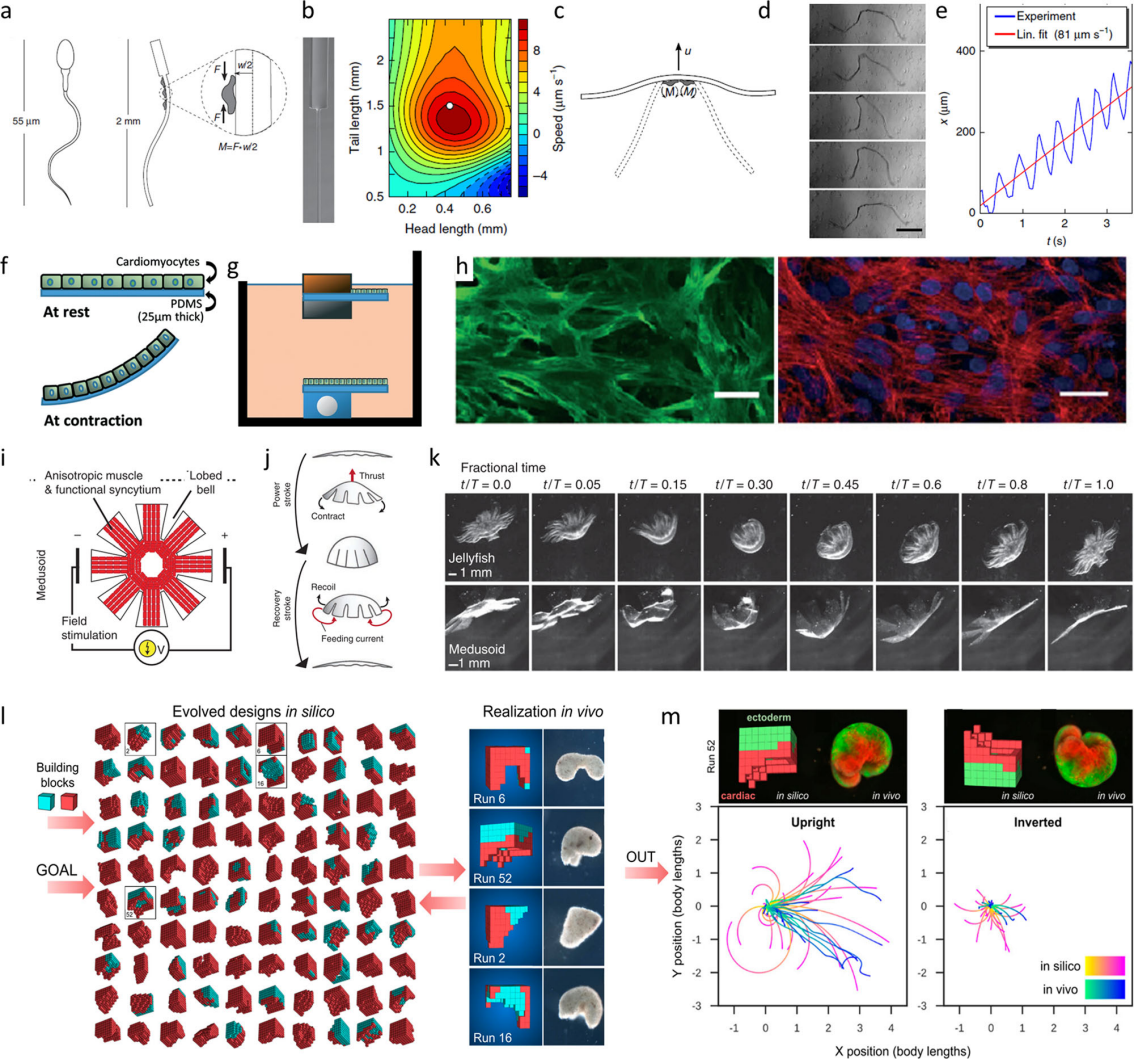
Recent trends in the development of cell seeded biorobotic systems. Concept design of a self-propelled biohybrid flagellum (right) with a similar motion to a spermatozoa (left) (**a**). Map of the predicted velocity as a function of head and tail dimensions of the biohybrid flagellum (**b**). Schematic of a two-tailed swimming biorobot (**c**). A sequence of images of the actuation of the two tailed swimmer (**d**) and the traveled distance and calculated velocity (**e**)^125^ (reprinted with permission from Nature Publishing Group, a division of Macmillan Publishers Limited: Nature Communications, A self-propelled biohybrid swimmer at low Reynolds number, Williams et al, Copyright 2014) Schematic diagram of the movement of a different biorobot, where the contraction of the cardiomyocyte bends the thin PDMS cantilever (**f**) of the floating or stationary biorobots (**g**). Immunostaining of cardiomyocyte marker, troponin-I (left) and actin cytoskeleton (right) show the growth of the cells without alignment (**h**)^64^. A tissue-engineered medusoid (**i**) with biomimetic jellyfish propulsion (**j**). Time lapse of a stroke cycle of a jellyfish and the medusoid (**k**)^130^ (reprinted with permission from Nature Publishing Group, a division of Macmillan Publishers Limited.: Springer Nature, Nature Biotechnology, A tissue-engineered jellyfish with biomimetic propulsion, Nawroth et al., Copyright 2012)) Artificial intelligence assisted design process of biorobots with predictable motion paths employing contractile (red) and passive (cyan) cell-based building blocks (l, left) as well as their in vivo realization using cardiomyocyte and epidermal cell progenitors (**l**, right). Predicted and in vivo movement of the designed models (**m**)^128^ (designing and manufacturing reconfigurable organisms and Transferal from silico to vivo by Kriegman et al. (CC BY 4.0)).

For cell seeding, usually primary rat cardiomyocytes are seeded on top of a substrate material, e.g. PDMS^64,125,128,130^. The substrate serves two functions: (i) it provides the cell adhesion and growth area and (ii) is an integral component of the robotic design that translates the cellular contraction into motion (Fig. 5). However, achieving a high level of biomechanical functionality is crucial. In current studies, the application of high cell densities, stimulation of cell alignment and myotube formation, or the mechanical design of the robotic elements were found to be adequate approaches to meet this demand. For forming aligned myotubes, versatile methods have been reported, e.g. by integrating materials with physical cues for cell guidance^49,94,132^, conditioning in bioreactors under static or cycling strain^14,16,55,56^, or electrical stimulation during conditioning^16,37^.

While early studies explored the general potential of biorobotic systems to enable movement in one direction (e.g. by contraction), recent work demonstrates that biorobots with multiple degrees of freedom can be fabricated. For instance, several groups created multi-directional biorobots by seeding cardiomyocytes on flexible PDMS substrates with isotropic geometries. The resulting biorobots were able to exercise bacteria and jellyfish-like movements^64,125,130^ (Fig. 5a–k). In this context, a very innovative approach is described by Kriegman and co-workers. Here, artificial intelligence algorithms are applied to design biorobotic systems with predictable motion paths^128^ (Fig. 5l, m).

### Biohybrid implants

Over 30 % of the human body weight is muscle tissue consisting of skeletal, heart and different other smooth muscles like abdominal muscles^53^. The muscle in the body has good self-healing properties from muscle stem cells and satellite cells, however, they can only repair small defects^103,133,134^. For the regeneration of defects with critical size caused e.g. by trauma or muscle dystrophy, traditionally muscle flaps from different regions of the body or by allogenic transplantation can be used with common disadvantages of donor site morbidity or immunogenic reactions^134,135^. To overcome these problems the biofabrication of muscle tissue implants gained attention.

Compared to the before-mentioned applications, muscle implants have the highest need for individualization and biological functionality. In order to match the complex anatomical architecture of native tissue, muscle implants require the orchestrated placement of different cell types and biomaterials with high spatial resolution^136^. Thus, mostly 3D-bioprinting strategies are applied as they offer the potential to generate multi-cellular structures with high spatial resolution^137^. The formation of functional myotubes as well as an intact innervation are of high relevance to ensure implant functionality and tissue integration^66,103^.

In order to maximize biological acceptance, as a cell source only human, ideally autologous patient-specific cells, can be used. Induced pluripotent stem cells theoretically offer an unlimited cell source for this application. They can be differentiated in all cell types required for muscle tissue fabrication: myoblasts, adipocytes, fibroblasts, and endothelial cells^138^. However, stem cell cultivation and expansion to yield sufficiently high cell numbers remain a challenge^113^.

**Fig. 6:**
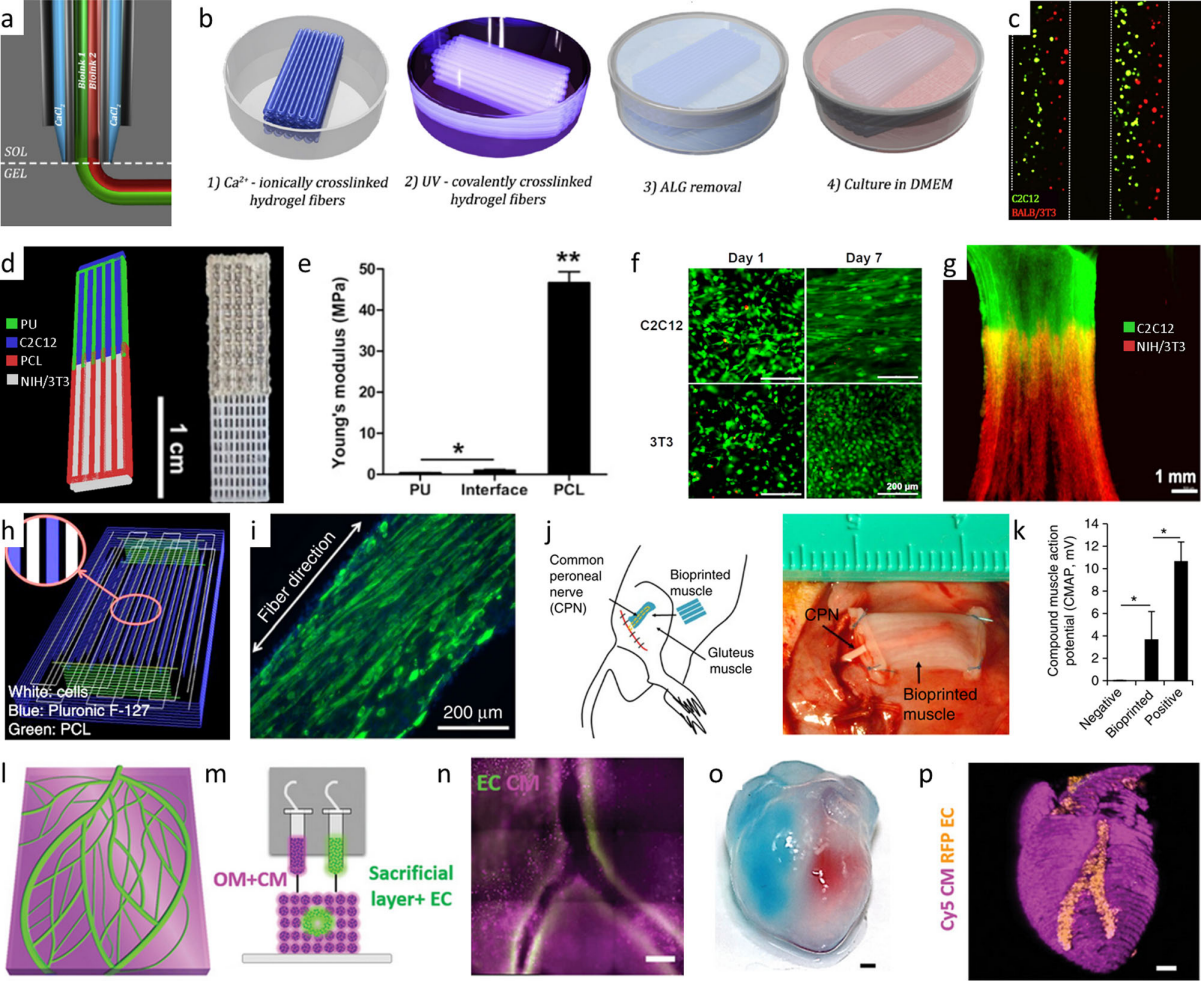
Examples of biofabricated muscle tissue for regenerative medicine. Coaxial extruder nozzle (**a**) used for the printing of high shape fidelity structures utilizing ionic and UV-based bioink crosslinking (**b**). Fluorescence image of the printed multicellular result comprising C2C12 muscle cells (green) and BALB/3T3 fibroblasts (red) (**c**)^58^ (3D bioprinting set-up and Multi-cellular 3D bioprinting through a microfluidic printing head by Costantini et al. (CC BY NC ND 4.0)) Bioprinted multimaterial muscle-tendon unit (**d**) showing different elastic moduli of the integrated scaffold materials of polyurethane (PU) and polycaprolactone (PCL) (**e**). The C2C12 muscle cells of the muscle-tendon unit show morphological changes in the fluorescence microscopic image into an elongated shape while NIH/3T3 fibroblasts keep their morphology (**f**). Fluorescently-labeled dual-cell printed constructs (green: DiO-labeled C2C12 cells; red: DiI-labeled NIH/ 3T3 cells; imaged at 7 d in culture) shows cell–cell interactions and cell migration (**g**)^54^ (© IOP Publishing. Reproduced with permission. All rights reserved.) Printing path of a fiber bundle design for muscle organization with PCL pillars and sacrificial Pluronic F-127 channels for nutrition and cell alignment (**h**). The cell alignment can be seen in the immunofluorescent staining for myosin heavy chain of the 3D printed muscle after 7 days differentiation (**i**). Subcutaneous implantation of the bioprinted muscle fiber bundle with host nerve integration (**j**). Assessment of the function of the bioprinted muscle construct after 4 weeks of implantation (positive control: the normal gastrocnemius muscle; negative control: the gluteus muscle after dissected common peroneal nerve) (**k**)^66^ (reprinted with permission from Nature Publishing Group, a division of Macmillan Publishers Limited: Nature Biotechnology, A 3D bioprinting system to produce human-scale tissue constructs with structural integrity, Kang et al, Copyright 2016) Printing of cardiac patches (**l**–**n**). Model of the cardiac patch (**l**) and a side view of the printing concept showing the sacrificial bioink with endothelial cells (EC) and the bioink made from decellularized omentum tissue (OM) with cardiomyocytes (CM) (**m**). Printed cardiac patch with cardiac tissue (actinin stained in pink) and blood vessels (CD31 stained in green) (**n**). Small scale human heart (**o**, **p**) printed in suspension to create hollow ventricles. The ventricles were filled with red and blue dye for visualization (**o**). The fluorescence image shows printed vasculature in the small-scale heart (CMs in pink, ECs in orange) (**p**)^17^ (© 2018 WILEY-VCH Verlag GmbH & Co. KGaA, Weinheim).

Besides cell choices, the applied matrix used for embedding the cells must also meet specific requirements^50^. It should be biologically active to allow adhesion, migration, proliferation, and maturation like the natural ECM^102^. For this reason, decellularized ECM hydrogels are often used as they offer an excellent environment for cell growth^17,24,50,139^. Furthermore, the employment of hydrogel blends can be observed^36,54,57,58^. Especially for 3D-bioprinting, these formulations improve shape fidelity and structural reinforcement, while maintaining high levels of biofunctionality. For example, combinations of GelMa^57^ or PEG-Fibrinogen^58^ with alginate have been described. While the gelatin and fibrinogen component foster biofunctionality, ionic crosslinking of the polysaccharide enables rapid gelation, higher printability and shape fidelity, as well as a more favorable mechanical microenvironment to promote proliferation and myotube formation^57^ (Fig. 6a–c).

Analogue to biorobotic systems, for the generation of functional muscle implants high cell densities and a high degree of cell alignment need to be achieved to ensure excellent biofunctionality of the tissue. In order to achieve this, in current research extrusion-based 3D-bioprinting methods are employed. The extruded bioink strands result in aligned polymer fibers that guide cell elongation parallel to the printing path^140^. Alternatively, contact guidance and mechanical reinforcement strategies have been applied to modulate cell alignment and the elastic modulus of the tissue^36,54,66^ (Fig. 6h, i).

In addition, muscle implants are thicker than muscle units in biorobotic systems, which can be supplied with nutrients by diffusion, and—in contrast to cultured meat—need to stay functional for prolonged times (months to years). For this reason, muscle implants require a high level of vascularization to actively support nutrient supply and maintain tissue functionality for prolonged periods^103^.

Tissues incorporating perfusable channels exhibited significantly higher cell viability in deeper regions than statically cultured counterparts^21^. In recent studies, this was achieved for example by co-axial extrusion of alginate and calcium chloride to create cell-lined nutritional channels^79^. Other groups employ sacrificial materials to create open lumen structures^17,36,66^. For instance, Noor and co-workers printed endothelial cell containing gelatin as sacrificial material in parallel with decellularized ECM bioinks supplemented with cardiomyocytes to create thick perfusable cardiac patches with cell-lined nutritional channels^17^ (Fig. 6l–n). Besides the top-down integration of channels by e.g. 3D-bioprinting, nutrient supply can be further improved by self-assembly of vascular structures. For instance, Lee and his colleagues employed a VEGF releasing, microporous scaffold to promote extrinsic vascularization^21^.

To further increase long-term clinical success, it is essential to recreate stem cell niches and provide neuronal integration. Stem cell niches offer a supply of undifferentiated stem cells which can replace muscle tissue cells on demand. Recreation of such niches were successfully reported, e.g. by employing collagen type I scaffolds coated with integrins and laminins with a stiffness of 1–2 kPa^70,71^. Other approaches focus on innervation to yield long-term functionality of the implant. Innervation was realized by inserting the host’s nerve into the biofabricated tissue (Fig. 6j, k). The formation of neuromuscular junctions indicate the high innervation potential of this approach^31,66^. Co-culturing human primary skeletal myoblasts and human-induced neural stem cells was demonstrated to be an exciting alternative route towards this goal^141,142^.

While the fabrication of longitudinal, skeletal muscle implants^36,66^ or heart muscle patches^143,144^ was mostly focused on in the past, recently, we observe a trend towards the fabrication of more complex geometries, such as heart ventricles^17,21,145,146^ (Fig. 6o, p). In order to provide structural integrity during printing, the concept of submerged or freeform reversible embedding of suspended hydrogels (FRESH) bioprinting is frequently applied in this context. Here, cell-laden hydrogels are printed or extruded into high-density support baths^147,148^ or a swollen hydrogel slurry^21^.

## Summary and outlook

Biofabrication of artificial muscles is an emerging field of research. With the advancement of biofabrication technologies, on the one hand, and its increasing range of applications covering cultured meat, biorobots, and biohybrid implants for regenerative medicine on the other hand, the investigation of muscle tissue fabrication methods gained particular attention in the recent decade. The biofabrication process generally comprises the tissue design (determination of requirements, desired function, and geometry, the bulk-material selection (cell types, matrix, and support materials, Chapter Physico-chemical composition of the bulk material), as well as strategies for spatio-temporal modification and subsequent conditioning of the generated structure into a biological tissue (Chapter Spatio-temporal modification). This review highlights the individual steps of the biofabrication process and provides a comprehensive overview on recent success stories for the above-mentioned fields of application.

As pointed out, cultured meat primarily has to be cost and resource efficient (Chapter Cultured meat). Our observations indicate that current fabrication processes are designed accordingly. Mostly simple bulk geometries are designed^12,112,113^, manual biofabrication methods with low spatio-temporal specification and texture, such as cell seeding^12,49,149^ or molding^13,108^, are applied, and the tissue is rarely conditioned. Furthermore, in most cases thin tissue slices were generated that rarely required supply channels, but could be readily provided with nutrients and oxygen by diffusion^11,12,150,151^. However, recent work stresses the importance of additional quality parameters, such as texture or mouthfeel, to increase consumer acceptance. For instance, this is accounted for by using anisotropic scaffolds with interconnected pores, produced e.g. by directed freeze drying, resulting in a preferential direction of cellular growth and thus texture^11,12^. Other studies texturize the muscle tissue by promoting cellular alignment via isometric strain^13,35^ and contact guidance^49,94^, or by co-cultivation with cells that exhibit equielastic ECM expression^11^.

Considering biorobotic systems, a high ultimate force to tissue volume ratio is the key performance indicator that determines the conditions of the fabrication process (Chapter Biorobotic systems). As for cultured meat, simple geometries and rather thin muscle sheets, which can be fed by diffusion, are employed^16,37,64,130^. However, in contrast to the former, high biofunctionality is required for prolonged time in this field of application. Thus bulk materials with high biological affinity, such as ECM-derived matrices, are mostly reported^14,16,55,56^. High force output is accomplished by promoting cell alignment either via isometric strain exerted by anchors^14,16,37^ or by smart scaffold designs with isotropic properties^64,125,130^.

In addition to the previously discussed fields of applications, bioartificially buil muscle tissue is also attracting increasing interest in the field of disease modeling and as a drug screening platform. In general, the same fabrication concepts and strategies as discussed in this article can be applied here. However, the latter demands in-depth understanding and comprehensive discussion of metabolic cell biological processes, pharmacokinetics, as well as disease mechanisms and therefore merits a dedicated representation in a separate review article^152^.

Finally, to bridge critical size defects following trauma or diseasebiohybrid muscle implants are becoming of age in regenerative medicine. In order to replace, support, or maintain its native counterpart, biofabricated muscle implants are subject to the most demanding requirements compared to the before-mentioned applications (Table 1). In particular, native architecture, prolonged functionality, and vascular integration of comparably large tissues need to be achieved. In this context, the application of more complex biofabrication methods, such as 3D-bioprinting, are frequently reported^17,21,36,66^. In order to match both high biofunctionality as well as shape fidelity, ECM-derived matrices blended with additional polymers, e.g. alginate, are used as matrix materials^57,58^. Interestingly, cell alignment is reported to be possible either by the inherent polymer chain orientation resulting from extrusion bioprinting^16,140^, by parallel or hybrid bioprinting of polymer strands for cell guiding^54^ or exertion of isometric strain^24,36,66^. Due to their size as well as the requirement to be connected to the native vascular system after implantation, the integration of vascular channels is inevitable for the production of muscle implants. In addition, to ensure that the vessels remain stable over time and do not occlude due to neoplasm formation or blood contact, lining with endothelial cells is required^17,21,79^. So far, this is mostly accomplished by post-fabrication cell seeding^79^ or the promotion of self-assembly processes^21^.

In summary, by comparing cultured meat, biorobotic systems, and muscle implants, we observe strong differences in the applied designs, cell types, matrices, and biofabrication processes. Both form and fabrication follow the function of the desired field of application as well as its most vital demands. For instance, even though biorobots and muscle implants share the same requirement in terms of pro-longed functionality and nutrient supply, due to their difference in size the importance of supply channel integration is only reasonable for muscle implants, so far. Instead, biorobots are most valuable when used in small scale. Compared to other actuators, such as electroactive polymers or pneumatic systems, biorobots impress with their high force to volume ratio^124,126^ and small size with only a fraction of a millimeter^128^. Technical systems usually require constant energy or pneumatic pressure supply to function. The advantage of biorobots is that their functional units, living cells, can be nourished by medium or soluble factors found in the blood. This aspect awards them an advantage over technical systems, especially for future in-vivo applications within the body.

For the future development of this exciting field of research, we observe already today highly interesting trends. The observed increase in more sophisticated design approaches, such as artificial intelligence^128^ or integrated neurons for targeted control of biorobots^55^, give us a taste of what previously unattainable functionalities will be enabled through the intelligent arrangement of cells, matrices, and supporting materials in the future. Similarly, mechanical properties and biohybrid reinforcement to foster cell maturation will become an even more prominent task, in order to improve the interaction of biological as well as mechanical parts and force transmission.

Thicker structures with fine texturing, marbled fat, and connective tissue that closely mimic nature will be the next milestone in the field of cultured meat. To accomplish this goal, we predict a change in the applied fabrication methods toward technologies that provide efficient and high-scale manufacturing at strongly increased spatial resolution. Novel 3D-bioprinting technologies and original methods will need to be developed for this task. In this context, today’s intricate muscle fabrication methods, such as those we observe in muscle implant manufacturing, offer important learning cases. The strategies currently used to manufacture muscle implants, such as bulk material modification for improved biological properties, printability and shape fidelity, will be transferred to increase the complexity of other applications, such as cultured meat or biorobotic systems. At the same time, muscle implant manufacturing will also benefit from the booming development of the other two fields of application. This holds true especially since implants combine a couple of key features that will become more prominent in both areas in the future, e.g. the high biofunctionality of biorobots and the aspired high volumes and texture of cultured meat. By solving the individual challenges and merging the knowhow of experts, synergetic leaps of innovation that inspire each other can be expected in all three fields of application, which so far are mostly viewed separately. The most recently presented study by Srinivasan and co-workers^153^ (Fig. 1b), which combines the concepts of biorobotic systems and biohybrid implants^153^, is a ground-breaking indicator for the unprecedented innovations and future trends that will be unleashed by the proposed technological fusion.

### Reporting summary

Further information on research design is available in the Nature Research Reporting Summary linked to this article.

## Supplementary information

Proof of Permissions

Reporting Summary
