# Supplementary material for: Recent trends in bioartificial muscle engineering and their applications in cultured meat, biorobotic systems and biohybrid implants
Source: Commun Biol. 2022 Jul 22;5:737. doi: 10.1038/s42003-022-03593-5 (PMC9307618; doi:10.1038/s42003-022-03593-5)
Supplement: Supplementary file 1 — Proof of Permissions [file 42003_2022_3593_MOESM1_ESM.zip › File 19 Holley 2016_RightsLink Printable License.pdf]

CCC Marketplace™

Order Confirmation

Thank you, your order has been placed. An email confirmation has been sent to you. Your order license details and printable licenses will be available within 24 hours. Please access Manage Account for final order details.

This is not an invoice. Please go to manage account to access your order history and invoices.

CUSTOMER INFORMATION

Payment by invoice: You can cancel your order until the invoice is generated by contacting customer service.

Billing Address

Eva Schätzlein
Technical University of Darmstadt
Karolinenplatz 5
Darmstadt, 64289
Germany

+49 61511622780
schaetzlein@idd.tu-darmstadt.de

Customer Location

Technical University of Darmstadt
Technical University of Darmstadt
Karolinenplatz 5
Darmstadt, 64289
Germany

PO Number (optional)

N/A

Payment options

Invoice

PENDING ORDER CONFIRMATION

Confirmation Number: Pending

Order Date: 04-Apr-2022

1. Lab on a chip

0,00 EUR

Article: Development and characterization of muscle-based actuators for self-stabilizing swimming biorobots.

**Order License ID**	Pending	**Publisher**	ROYAL SOCIETY OF CHEMISTRY
**ISSN**	1473-0197		
**Type of Use**	Republish in a journal/magazine	**Portion**	Image/photo /illustration

LICENSED CONTENT

**Publication Title**	Lab on a chip	**Rightsholder**	Royal Society of Chemistry
**Article Title**	Development and characterization of muscle-based actuators for self-stabilizing swimming biorobots.	**Publication Type**	Journal
		**Start Page**	3473
		**End Page**	3484
**Author/Editor**	Royal Society of Chemistry (Great Britain)	**Issue**	18
		**Volume**	16
**Date**	01/01/2001		
**Language**	English		
**Country**	United Kingdom of Great Britain and Northern Ireland		

REQUEST DETAILS

**Portion Type**	Image/photo/illustration	**Distribution**	Worldwide
**Number of images / photos / illustrations**	2	**Translation**	Original language of publication
**Format (select all that apply)**	Print, Electronic	**Copies for the disabled?**	No
**Who will republish the content?**	Publisher, STM	**Minor editing privileges?**	Yes
**Duration of Use**	Life of current edition	**Incidental promotional use?**	No
**Lifetime Unit Quantity**	More than 2,000,000	**Currency**	EUR
**Rights Requested**	Main product		

NEW WORK DETAILS

**Title**	Recent trends in bioartificial muscle engineering and their applications in cultured meat, biorobotic systems and biohybrid implants	**Publisher imprint**	Springer Nature
		**Expected publication date**	2022-04-30
		**Expected size (number of pages)**	28
**Author**	Eva Schätzlein, Andreas Blaeser	**Standard identifier**	N/A
**Publication**	Communications Biology		
**Publisher**	Springer Nature		

ADDITIONAL DETAILS

**Order reference number**	N/A	**The requesting person / organization to appear on the license**	Technical University of Darmstadt

REUSE CONTENT DETAILS

**Title, description or numeric reference of the portion(s)**	Figure 1, Figure 5	**Title of the article/chapter the portion is from**	Development and characterization of muscle-based actuators for self-stabilizing swimming biorobots.
**Editor of portion(s)**	Holley, Merrel T.; Nagarajan, Neerajha; Danielson, Christian; Zorlutuna, Pinar; Park, Kidong	**Author of portion(s)**	Holley, Merrel T.; Nagarajan, Neerajha; Danielson, Christian; Zorlutuna, Pinar; Park, Kidong
**Volume of serial or monograph**	16	**Issue, if republishing an article from a serial**	18
**Page or page range of portion**	3473-3484	**Publication date of portion**	2016-09-21

Total Items: 1

Total Due: 0,00 EUR

Accepted: All Publisher and CCC Terms and Conditions
