# Supplementary material for: Recent trends in bioartificial muscle engineering and their applications in cultured meat, biorobotic systems and biohybrid implants
Source: Commun Biol. 2022 Jul 22;5:737. doi: 10.1038/s42003-022-03593-5 (PMC9307618; doi:10.1038/s42003-022-03593-5)
Supplement: Supplementary file 1 — Proof of Permissions [file 42003_2022_3593_MOESM1_ESM.zip › File 23 ITOP Publishing, Ltd Terms and Conditions.pdf]

IOP Publishing, Ltd Terms and Conditions

These special terms and conditions are in addition to the standard terms and conditions for CCC's Republication Service and, together with those standard terms and conditions, govern the use of the Works. As the User you will make all reasonable efforts to contact the author(s) of the article which the Work is to be reused from, to seek consent for your intended use. Contacting one author who is acting expressly as authorised agent for their co-author(s) is acceptable. User will reproduce the following wording prominently alongside the Work: the source of the Work, including author, article title, title of journal, volume number, issue number (if relevant), page range (or first page if this is the only information available) and date of first publication; and a link back to the article (via DOI); and if practicable, and IN ALL CASES for new works published under any of the Creative Commons licences, the words "© IOP Publishing. Reproduced with permission. All rights reserved" Without the express permission of the author(s) and the Rightsholder of the article from which the Work is to be reused, User shall not use it in any way which, in the opinion of the Rightsholder, could: (i) distort or alter the author(s)' original intention(s) and meaning; (ii) be prejudicial to the honour or reputation of the author(s); and/or (iii) imply endorsement by the author(s) and/or the Rightsholder. This licence does not apply to any article which is credited to another source and which does not have the copyright line '© IOP Publishing Ltd'. User must check the copyright line of the article from which the Work is to be reused to check that IOP Publishing Ltd has all the necessary rights to be able to grant permission. User is solely responsible for identifying and obtaining separate licences and permissions from the copyright owner for reuse of any such third party material/figures which the Rightsholder is not the copyright owner of. The Rightsholder shall not reimburse any fees which User pays for a republication license for such third party content. This licence does not apply to any material/figure which is credited to another source in the Rightsholder's publication or has been obtained from a third party. User must check the Version of Record of the article from which the Work is to be reused, to check whether any of the material in the Work is third party material. Third party citations and/or copyright notices and/or permissions statements may not be included in any other version of the article from which the Work is to be reused and so cannot be relied upon by the User. User is solely responsible for identifying and obtaining separate licences and permissions from the copyright owner for reuse of any such third party material/figures where the Rightsholder is not the copyright owner. The Rightsholder shall not reimburse any fees which User pays for a republication license for such third party content. User and CCC acknowledge that the Rightsholder may, from time to time, make changes or additions to these special terms and conditions without express notification, provided that these shall not apply to permissions already secured and paid for by User prior to such change or addition. User acknowledges that the Rightsholder (which includes companies within its group and third parties for whom it publishes its titles) may make use of personal data collected through the service in the course of their business. If User is the author of the Work, User may automatically have the right to reuse it under the rights granted back when User transferred the copyright in the article to the Rightsholder. User should check the copyright form and the relevant author rights policy to check whether permission is required. If User is the author of the Work and does require permission for proposed reuse of the Work, User should select 'Author of requested content' as the Requestor Type. The Rightsholder shall not reimburse any fees which User pays for a republication license. If User is the author of the article which User wishes to reuse in User's thesis or dissertation, the republication licence covers the right to include the Version of Record of the article, provided it is not then shared or deposited online. User must include citation details. Where User wishes to share their thesis or dissertation online, they should remove the Version of Record before uploading it. User may include a Preprint or the Accepted Manuscript (after the embargo period) in the online version of the thesis or dissertation, provided they do so in accordance with the Rightsholder's policies on sharing Preprints or Accepted Manuscripts. User may need to obtain separate permission for any third party content included within the article. User must check this with the copyright owner of such third party content. Any online or commercial use of User's thesis or dissertation containing the article, including publication via ProQuest, would need to be expressly notified in writing to the Rightsholder at the time of request and would require separate written permission from the Rightsholder. As well as CCC, the Rightsholder shall have the right to bring any legal action that it deems necessary to enforce its rights should it consider that the Work infringes those rights in any way. For content reuse requests that qualify for permission under the STM Permissions Guidelines, which may be updated from time to time, the STM Permissions Guidelines supplement the terms and conditions contained in this license.
