# Supplementary material for: Recent trends in bioartificial muscle engineering and their applications in cultured meat, biorobotic systems and biohybrid implants
Source: Commun Biol. 2022 Jul 22;5:737. doi: 10.1038/s42003-022-03593-5 (PMC9307618; doi:10.1038/s42003-022-03593-5)
Supplement: Supplementary file 1 — Proof of Permissions [file 42003_2022_3593_MOESM1_ESM.zip › File 9 Post 2014_RightsLink Printable License.pdf]

ELSEVIER ORDER DETAILS

Apr 04, 2022

Order Number	501722600
Order date	Apr 04, 2022
Licensed Content Publisher	Elsevier
Licensed Content Publication	Elsevier Books
Licensed Content Title	Principles of Tissue Engineering
Licensed Content Author	Mark Post,Cor van der Weele
Licensed Content Date	2014
Licensed Content Pages	16
Start Page	1647
End Page	1662
Type of Use	reuse in a journal/magazine
Requestor type	publisher
Portion	figures/tables/illustrations

Number of figures/tables /illustrations	1
Format	both print and electronic
Are you the author of this Elsevier chapter?	No
Will you be translating?	No
Title of new article	Recent trends in bioartificial muscle engineering and their applications in cultured meat, biorobotic systems and biohybrid implants
Lead author	Eva Schätzlein
Title of targeted journal	Communications Biology
Publisher	Springer Nature
Expected publication date	May 2022
Portions	Figure 78.2
Requestor Location	Technical University of Darmstadt<br>Technical University of Darmstadt<br><br>Karolinenplatz 5<br>Darmstadt, 64289<br>Germany<br>Attn: Technical University of Darmstadt
Publisher Tax ID	GB 494 6272 12
Total	Not Available
