# Supplementary material for: Recent trends in bioartificial muscle engineering and their applications in cultured meat, biorobotic systems and biohybrid implants
Source: Commun Biol. 2022 Jul 22;5:737. doi: 10.1038/s42003-022-03593-5 (PMC9307618; doi:10.1038/s42003-022-03593-5)
Supplement: Supplementary file 1 — Proof of Permissions [file 42003_2022_3593_MOESM1_ESM.zip › Permissions Overview COMMSBIO-21-1864B.docx]

# Proof of permissions for reused images: COMMSBIO-21-1864B

| File number | First Author Name, Publication Year | Source | Licence | Attributed in manuscript | Image |
| --- | --- | --- | --- | --- | --- |
| 1 | Kang 2021 | ^1^  Figure 5c | CC BY 4.0 | yes | 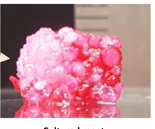 |
| 2 | Lui 2017 | ^2^  Figure 3 | CC BY 4.0 | yes | 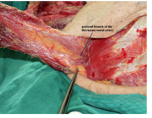 |
| 3 | Blaeser 2013 | ^3^  Figure 2 A | CC BY 4.0 | yes | 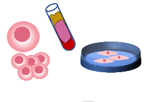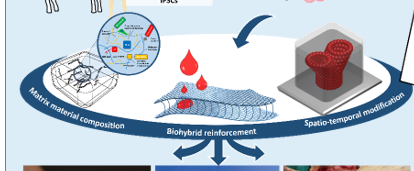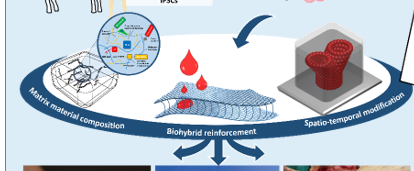 |
| 4 | Srinivasan 2021 | ^4^  Figure 1 Figure 2 | See Attachment  RightsLink Printable License | yes | 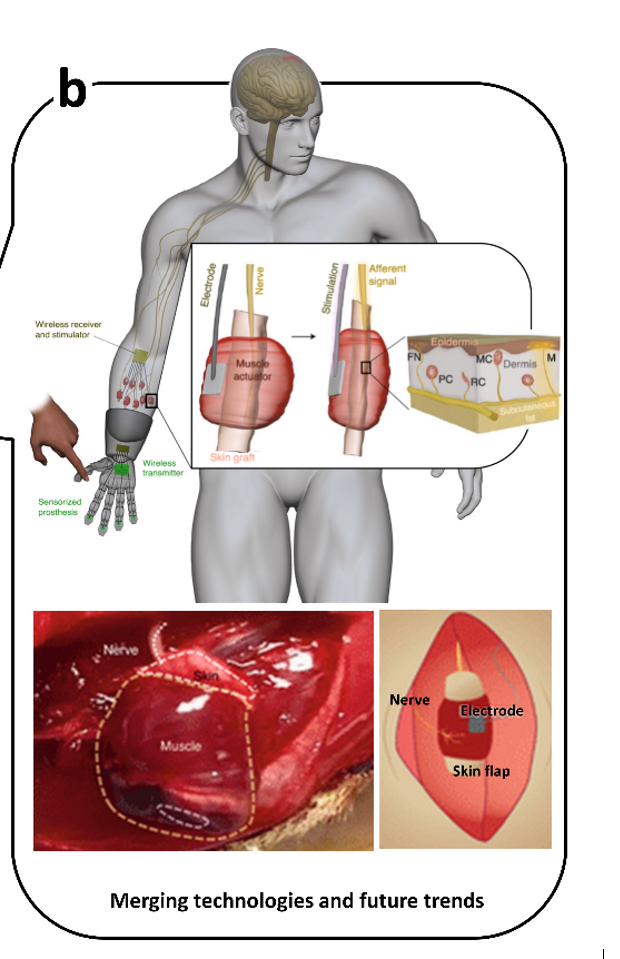 |
| 5 | Lee 2019 | ^5^  Figure 2 C, E, F, M, N | See Attachment  RightsLink Printable License | yes | 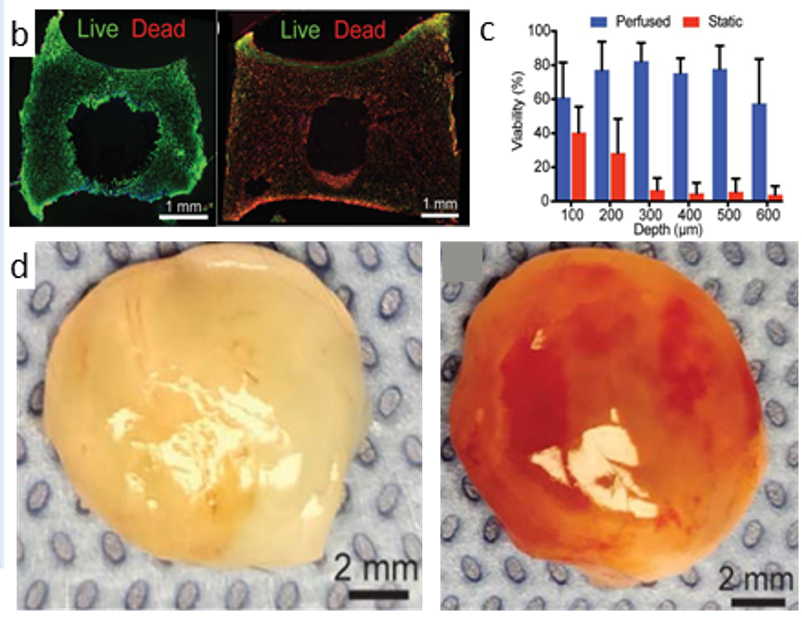 |
| 6 | Xie 2019 | ^6^  Figure 1B | CC BY 4.0 | yes | 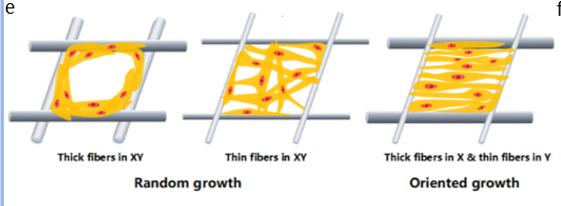 |
| 7 | Schäfer 2020 | ^7^  Scheme 1  Figure 1 D  Figure 3 F | CC BY 4.0 | yes | 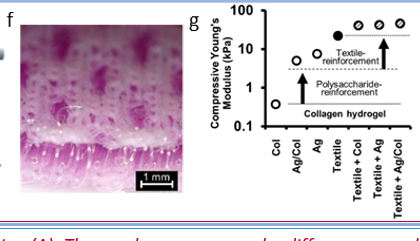  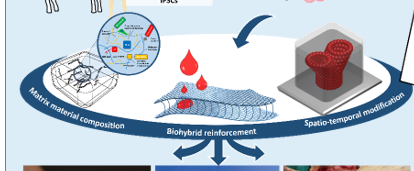 |
| 8 | MacQueen 2019 | ^8^  Figure 1 and 3 | CC BY 4.0 | yes | 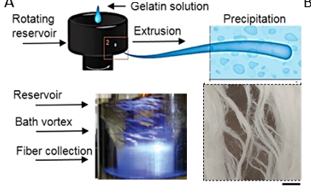 |
| 9 | Post 2014 | ^9^  Figure 78.2 | See Attachment  RightsLink Printable License | yes | 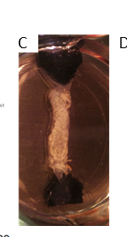 |
| 10 | Zidaric 2020 | ^10^  Figure 4 | See Attachment  RightsLink Printable License | yes | 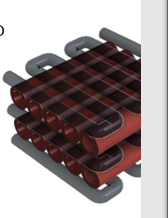 |
| 11 | Ben-Arye 2020 | ^11^  Figure 1  Figure 7 | See Attachment  RightsLink Printable License | yes | 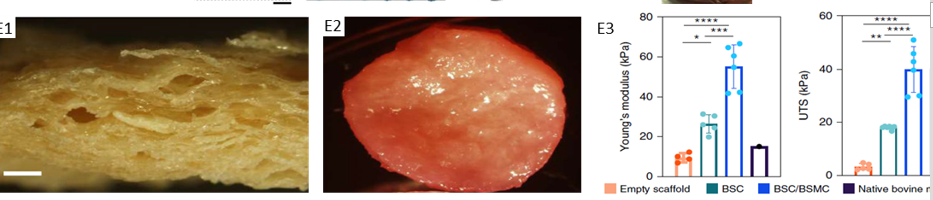 |
| 12 | Furuhashi 2021 | ^12^  Figure 1 b  Figure 2a  Figure 3 e  Figure 4 b | CC BY 4.0 | yes | 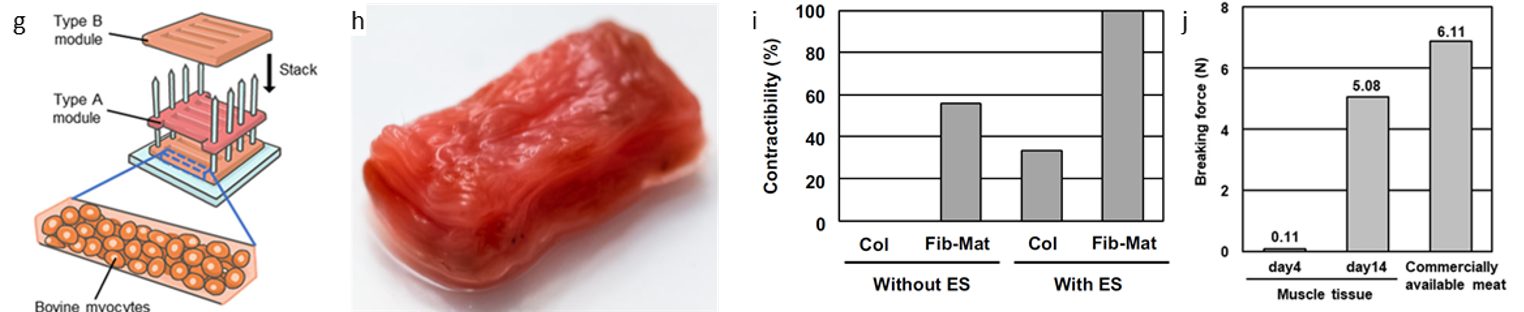 |
| 13 | Simsa 2019 | ^13^  Figure 3 A  Figure 9 | CC BY 4.0 | yes | 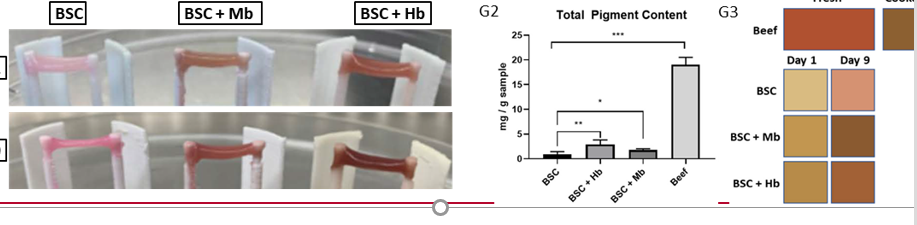 |
| 14 | Mestre 2018 | ^14^  Figure 1 D, Figure 2 A, G  Figure 5 B | See Attachment  RightsLink Printable License | yes | 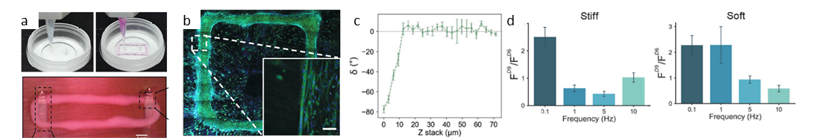 |
| 15 | Raman 2016 | ^15^  Figure 1 a, d  Figure 3 a, b, c | See Attached Email PNAS Permissions | no | 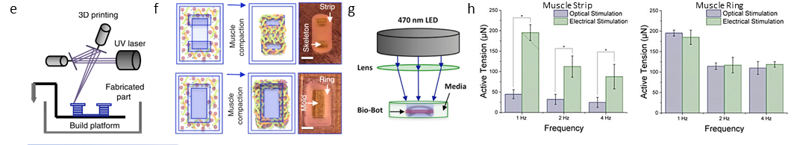 |
| 16 | Morimoto 2018 | ^16^  Figure 1 Figure 2 Figure 3 Figure 4 | See Attachment  RightsLink Printable License | yes | 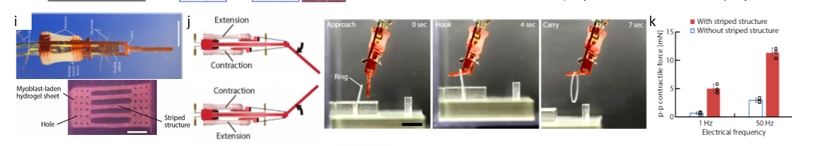  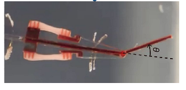 |
| 17 | Aydin 2019 | ^17^  Figure 2 a,b, d, e  Figure 4 a | CC BY-NC-ND | yes | 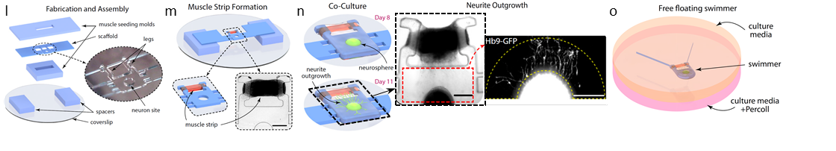 |
| 18 | Williams 2014 | ^18^  Figure 1 a, b, c  Figure 2 d  Figure 6 a, b, c | See Attachment  RightsLink Printable License | yes | 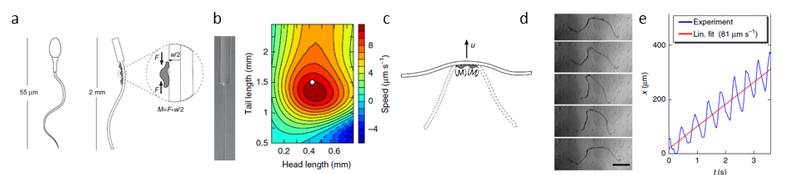 |
| 19 | Holley 2016 | ^19^  Figure 1  Figure 5 | See Attachment  RightsLink Printable License | no | 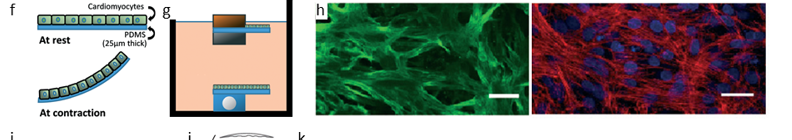 |
| 20 | Nawroth 2012 | ^20^  Figure 1 a,b  Figure 2 a | See Attachment  RightsLink Printable License | yes | 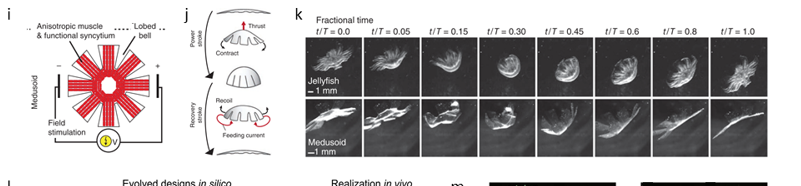 |
| 21 | Kriegman 2020 | ^21^  Figure 1  Figure 4 | CC BY 4.0 | yes | 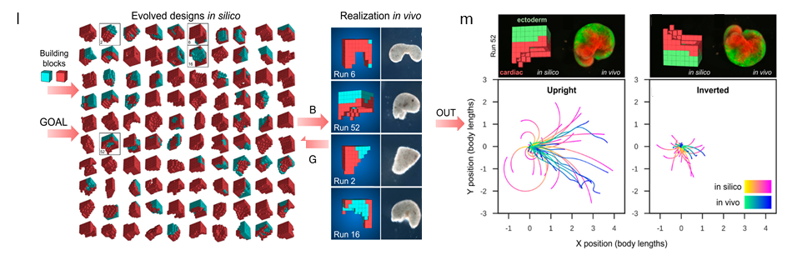 |
| 22 | Costantini 2017 | ^22^  Figure 1 c, d  Figure 8 b | CC BY-NC-ND 4.0  See Attachment  RightsLink Printable License | yes | 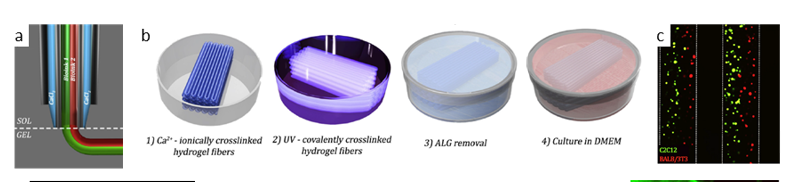 |
| 23 | Merceron 2017 | ^23^  Figure 2  Figure 3  Figure 4  Figure 5 | See Attachment  ITOP Publishing, Ltd Terms and Conditions | yes | 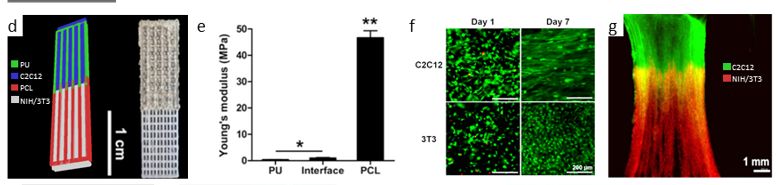 |
| 24 | Kang 2016 | ^24^  Figure 6 b, g, h, i, n | See Attachment  RightsLink Printable License | yes | 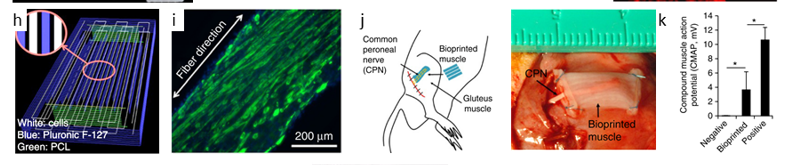 |
| 25 | Noor 2019 | ^25^  Figure 4 a, b, f,  Figure 6 h, i | CC BY 4.0 | yes | 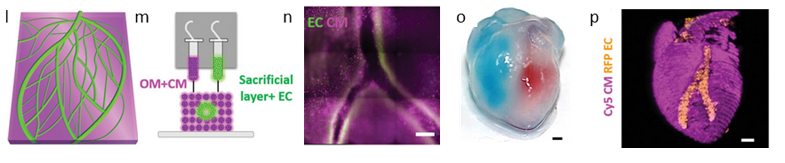 |

References

1. Kang, D.-H. *et al.* Engineered whole cut meat-like tissue by the assembly of cell fibers using tendon-gel integrated bioprinting. *Nat. Commun.* 12, 5059 (2021).

2. Liu, M., Liu, W., Yang, X., Guo, H. & Peng, H. Pectoralis Major Myocutaneous Flap for Head and Neck Defects in the Era of Free Flaps: Harvesting Technique and Indications. *Sci. Rep.* 7, 1–9 (2017).

3. Blaeser, A. *et al.* Biofabrication Under Fluorocarbon: A Novel Freeform Fabrication Technique to Generate High Aspect Ratio Tissue-Engineered Constructs. *Biores. Open Access* 2, 374–384 (2013).

4. S. Srinivasan, S. & M. Herr, H. A cutaneous mechanoneural interface for neuroprosthetic feedback. *Nat. Biomed. Eng.* (2021) doi:10.1038/s41551-020-00669-7.

5. Lee, A. *et al.* 3D bioprinting of collagen to rebuild components of the human heart. *Science (80-. ).* 365, 482–487 (2019).

6. Xie, C. *et al.* Structure-induced cell growth by 3D printing of heterogeneous scaffolds with ultrafine fibers. *Mater. Des.* 181, 1–11 (2019).

7. Schäfer, B. *et al.* Warp-Knitted Spacer Fabrics: A Versatile Platform to Generate Fiber-Reinforced Hydrogels for 3D Tissue Engineering. *Materials (Basel).* 13, 3518 (2020).

8. MacQueen, L. A. *et al.* Muscle tissue engineering in fibrous gelatin: implications for meat analogs. *npj Sci. Food* 3, 1–12 (2019).

9. Post, M. J. & van der Weele, C. *Principles of Tissue Engineering for Food*. *Principles of Tissue Engineering: Fourth Edition* (Elsevier, 2013). doi:10.1016/B978-0-12-398358-9.00078-1.

10. Zidarič, T., Milojević, M., Vajda, J., Vihar, B. & Maver, U. Cultured Meat: Meat Industry Hand in Hand with Biomedical Production Methods. *Food Eng. Rev.* (2020) doi:10.1007/s12393-020-09253-w.

11. Ben-Arye, T. *et al.* Textured soy protein scaffolds enable the generation of three-dimensional bovine skeletal muscle tissue for cell-based meat. *Nat. Food* 1, 210–220 (2020).

12. Furuhashi, M. *et al.* Formation of contractile 3D bovine muscle tissue for construction of millimetre-thick cultured steak. *npj Sci. Food* 5, 1–8 (2021).

13. Simsa, R. *et al.* Extracellular heme proteins influence bovine myosatellite cell proliferation and the color of cell-based meat. *Foods* 8, (2019).

14. Mestre, R. *et al.* Force Modulation and Adaptability of 3D‐Bioprinted Biological Actuators Based on Skeletal Muscle Tissue. *Adv. Mater. Technol.* 4, 1800631 (2018).

15. Raman, R. *et al.* Optogenetic skeletal muscle-powered adaptive biological machines. *Proc. Natl. Acad. Sci. U. S. A.* 113, 3497–3502 (2016).

16. Morimoto, Y., Onoe, H. & Takeuchi, S. Biohybrid robot powered by an antagonistic pair of skeletal muscle tissues. *Sci. Robot.* 3, 1–11 (2018).

17. Aydin, O. *et al.* Neuromuscular actuation of biohybrid motile bots. *Proc. Natl. Acad. Sci. U. S. A.* 116, 19841–19847 (2019).

18. Williams, B. J., Anand, S. V., Rajagopalan, J. & Saif, M. T. A. A self-propelled biohybrid swimmer at low Reynolds number. *Nat. Commun.* 5, 1–8 (2014).

19. Holley, M. T., Nagarajan, N., Danielson, C., Zorlutuna, P. & Park, K. Development and characterization of muscle-based actuators for self-stabilizing swimming biorobots. *Lab Chip* 16, 3473–3484 (2016).

20. Nawroth, J. C. *et al.* A tissue-engineered jellyfish with biomimetic propulsion. *Nat. Biotechnol.* 30, 792–797 (2012).

21. Kriegman, S., Blackiston, D., Levin, M. & Bongard, J. A scalable pipeline for designing reconfigurable organisms. *Proc. Natl. Acad. Sci. U. S. A.* 117, 1853–1859 (2020).

22. Costantini, M. *et al.* Microfluidic-enhanced 3D bioprinting of aligned myoblast-laden hydrogels leads to functionally organized myofibers in vitro and in vivo. *Biomaterials* 131, 98–110 (2017).

23. Merceron, T. K. *et al.* A 3D bioprinted complex structure for engineering the muscle-tendon unit. *Biofabrication* 7, (2015).

24. Kang, H. W. *et al.* A 3D bioprinting system to produce human-scale tissue constructs with structural integrity. *Nat. Biotechnol.* 34, 312–319 (2016).

25. Noor, N. *et al.* 3D Printing of Personalized Thick and Perfusable Cardiac Patches and Hearts. *Adv. Sci.* 6, (2019).
