# Supplementary material for: Recent trends in bioartificial muscle engineering and their applications in cultured meat, biorobotic systems and biohybrid implants
Source: Commun Biol. 2022 Jul 22;5:737. doi: 10.1038/s42003-022-03593-5 (PMC9307618; doi:10.1038/s42003-022-03593-5)
Supplement: Supplementary file 1 — Proof of Permissions [file 42003_2022_3593_MOESM1_ESM.zip › Permissions Overview COMMSBIO-21-1864B.pdf]

Proof of permissions for reused images: COMMSBIO-21-1864B

File number	First Author Name, Publication Year	Source	Licence	Attributed in manuscript	Image
1	Kang 2021	[1] Figure 5c	CC BY 4.0	yes	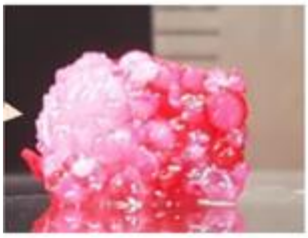
2	Lui 2017	[2] Figure 3	CC BY 4.0	yes	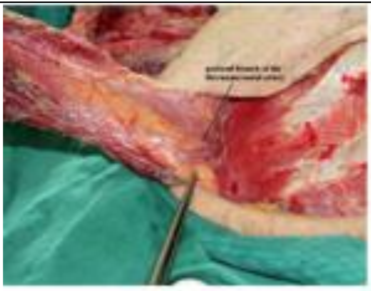
3	Blaeser 2013	[3] Figure 2 A	CC BY 4.0	yes	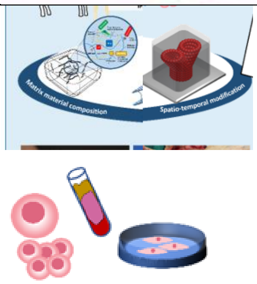
4	Srinivasan 2021	[4] Figure 1 Figure 2	See Attachment RightsLink Printable License	yes	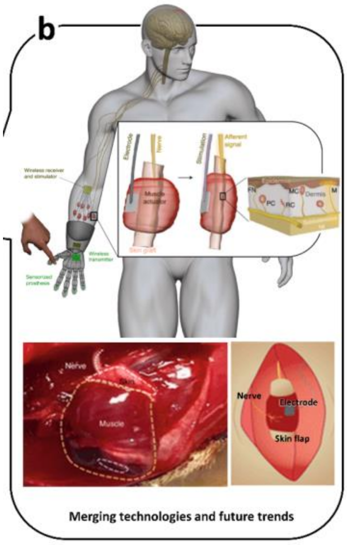
5	Lee 2019	[5] Figure 2 C, E, F, M, N	See Attachment RightsLink Printable License	yes	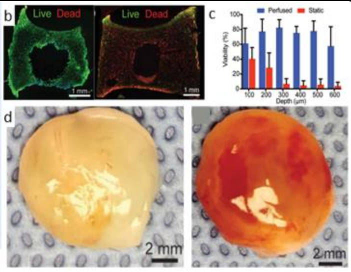

6	Xie 2019	[6] Figure 1B	CC BY 4.0	yes	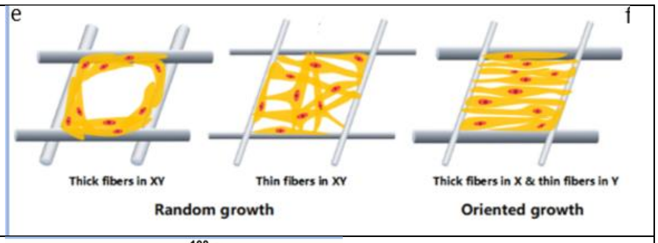
7	Schäfer 2020	[7] Scheme 1 Figure 1 D Figure 3 F	CC BY 4.0	yes	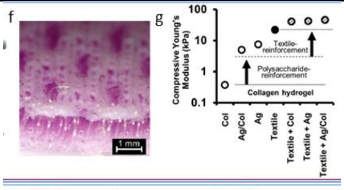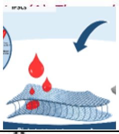
8	MacQueen 2019	[8] Figure 1 and 3	CC BY 4.0	yes	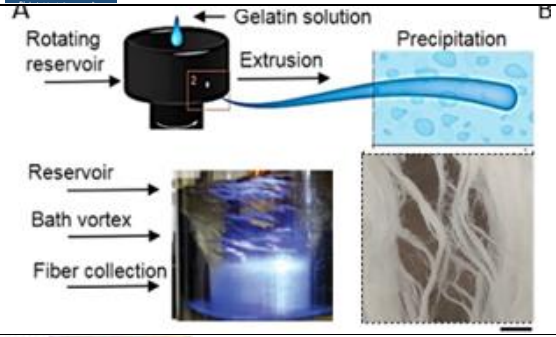
9	Post 2014	[9] Figure 78.2	See Attachment RightsLink Printable License	yes	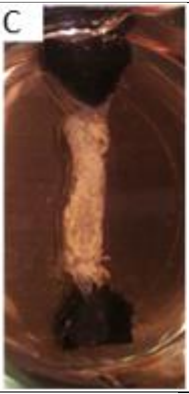
10	Zidaric 2020	[10] Figure 4	See Attachment RightsLink Printable License	yes	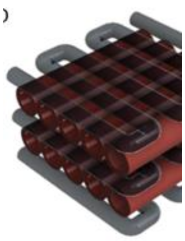
11	Ben-Arye 2020	[11] Figure 1 Figure 7	See Attachment RightsLink Printable License	yes	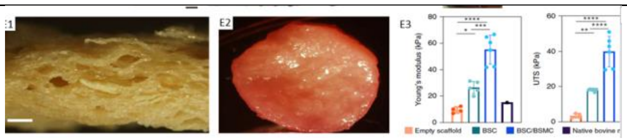
12	Furuhashi 2021	[12] Figure 1 b Figure 2a Figure 3 e Figure 4 b	CC BY 4.0	yes	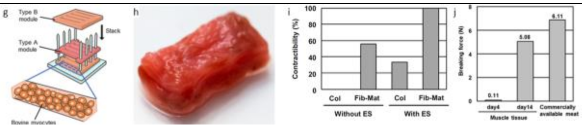

13	Simsa 2019	13<br>Figure 3 A<br>Figure 9	CC BY 4.0	yes	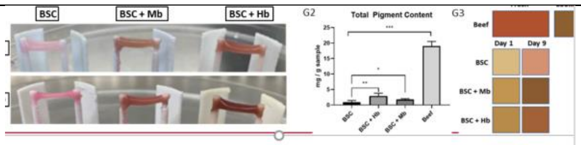
14	Mestre 2018	14<br>Figure 1 D,<br>Figure 2 A, G<br>Figure 5 B	See Attachment RightsLink Printable License	yes	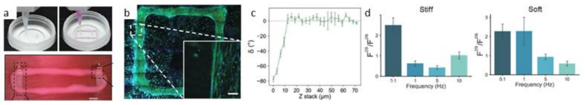
15	Raman 2016	15<br>Figure 1 a, d<br>Figure 3 a, b, c	See Attached Email PNAS Permissions	no	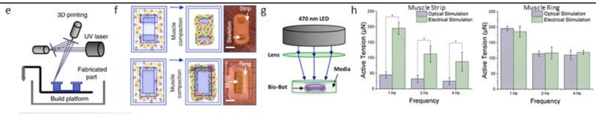
16	Morimoto 2018	16<br>Figure 1<br>Figure 2<br>Figure 3<br>Figure 4	See Attachment RightsLink Printable License	yes	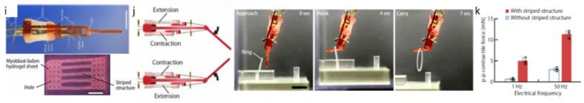 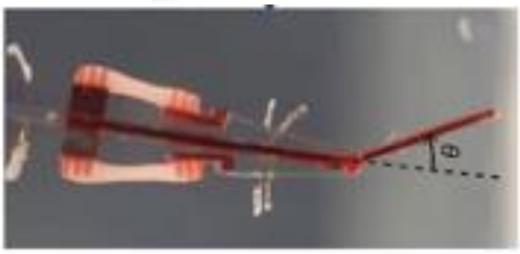
17	Aydin 2019	17<br>Figure 2 a,b, d, e<br>Figure 4 a	CC BY-NC-ND	yes	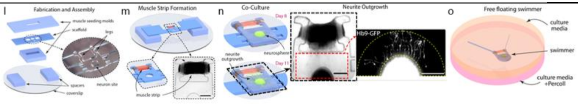
18	Williams 2014	18<br>Figure 1 a, b, c<br>Figure 2 d<br>Figure 6 a, b, c	See Attachment RightsLink Printable License	yes	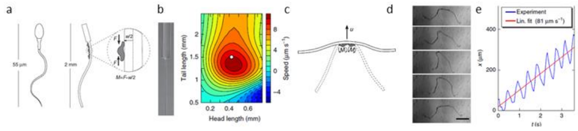
19	Holley 2016	19<br>Figure 1<br>Figure 5	See Attachment RightsLink Printable License	no	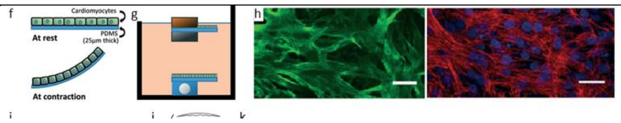
20	Nawroth 2012	20<br>Figure 1 a,b<br>Figure 2 a	See Attachment RightsLink Printable License	yes	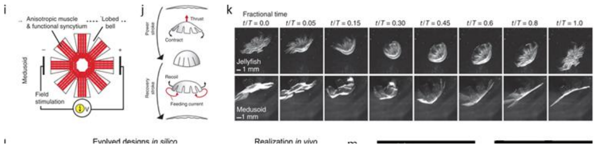
21	Kriegman 2020	21<br>Figure 1<br>Figure 4	CC BY 4.0	yes	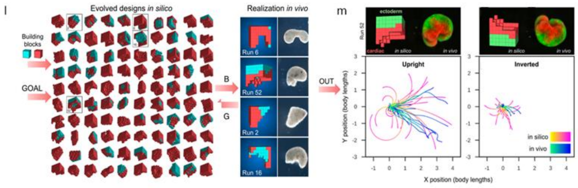
22	Costantini 2017	22<br>Figure 1 c, d<br>Figure 8 b	CC BY-NC-ND 4.0<br>See Attachment	yes	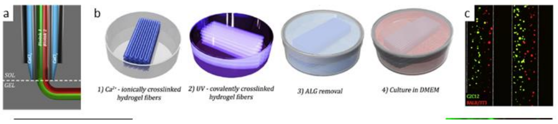

			RightsLink Printable License		
23	Merceron 2017	[23] Figure 2 Figure 3 Figure 4 Figure 5	See Attachment ITOP Publishing, Ltd Terms and Conditions	yes	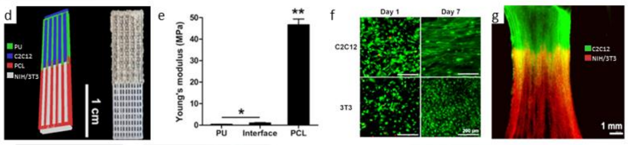
24	Kang 2016	[24] Figure 6 b, g, h, i, n	See Attachment RightsLink Printable License	yes	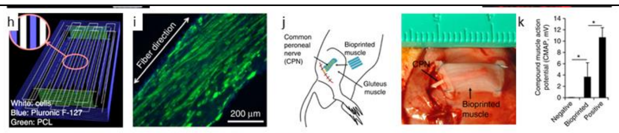
25	Noor 2019	[25] Figure 4 a, b, f, Figure 6 h, i	CC BY 4.0	yes	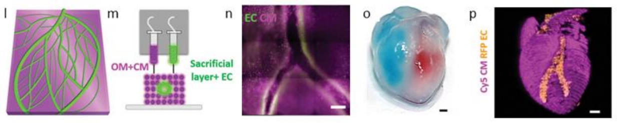

References

1. Kang, D.-H. *et al.* Engineered whole cut meat-like tissue by the assembly of cell fibers using tendon-gel integrated bioprinting. *Nat. Commun.* 12, 5059 (2021).

2. Liu, M., Liu, W., Yang, X., Guo, H. & Peng, H. Pectoralis Major Myocutaneous Flap for Head and Neck Defects in the Era of Free Flaps: Harvesting Technique and Indications. *Sci. Rep.* 7, 1–9 (2017).

3. Blaeser, A. *et al.* Biofabrication Under Fluorocarbon: A Novel Freeform Fabrication Technique to Generate High Aspect Ratio Tissue-Engineered Constructs. *Biores. Open Access* 2, 374–384 (2013).

4. S. Srinivasan, S. & M. Herr, H. A cutaneous mechanoneural interface for neuroprosthetic feedback. *Nat. Biomed. Eng.* (2021) doi:10.1038/s41551-020-00669-7.

5. Lee, A. *et al.* 3D bioprinting of collagen to rebuild components of the human heart. *Science (80-.).* 365, 482–487 (2019).

6. Xie, C. *et al.* Structure-induced cell growth by 3D printing of heterogeneous scaffolds with ultrafine fibers. *Mater. Des.* 181, 1–11 (2019).

7. Schäfer, B. *et al.* Warp-Knitted Spacer Fabrics: A Versatile Platform to Generate Fiber-Reinforced Hydrogels for 3D Tissue Engineering. *Materials (Basel).* 13, 3518 (2020).

8. MacQueen, L. A. *et al.* Muscle tissue engineering in fibrous gelatin: implications for meat analogs. *npj Sci. Food* 3, 1–12 (2019).

9. Post, M. J. & van der Weele, C. *Principles of Tissue Engineering for Food*. *Principles of Tissue Engineering: Fourth Edition* (Elsevier, 2013). doi:10.1016/B978-0-12-398358-9.00078-1.

10. Zidarič, T., Milojević, M., Vajda, J., Vihar, B. & Maver, U. Cultured Meat: Meat Industry Hand in Hand with Biomedical Production Methods. *Food Eng. Rev.* (2020) doi:10.1007/s12393-020-09253-w.

11. Ben-Arye, T. *et al.* Textured soy protein scaffolds enable the generation of three-dimensional bovine skeletal muscle tissue for cell-based meat. *Nat. Food* 1, 210–220 (2020).

12. Furuhashi, M. *et al.* Formation of contractile 3D bovine muscle tissue for construction of millimetre-thick cultured steak. *npj Sci. Food* 5, 1–8 (2021).

13. Simsa, R. *et al.* Extracellular heme proteins influence bovine myosatellite cell proliferation and the color of cell-based meat. *Foods* 8, (2019).

14. Mestre, R. *et al.* Force Modulation and Adaptability of 3D-Bioprinted Biological Actuators Based on Skeletal Muscle Tissue. *Adv. Mater. Technol.* 4, 1800631 (2018).

15. Raman, R. *et al.* Optogenetic skeletal muscle-powered adaptive biological machines. *Proc. Natl. Acad. Sci. U. S. A.* 113, 3497–3502 (2016).

16. Morimoto, Y., Onoe, H. & Takeuchi, S. Biohybrid robot powered by an antagonistic pair of skeletal muscle tissues. *Sci. Robot.* 3, 1–11 (2018).

17. Aydin, O. *et al.* Neuromuscular actuation of biohybrid motile bots. *Proc. Natl. Acad. Sci. U. S. A.* 116, 19841–19847 (2019).

18. Williams, B. J., Anand, S. V., Rajagopalan, J. & Saif, M. T. A. A self-propelled biohybrid swimmer at low Reynolds number. *Nat. Commun.* 5, 1–8 (2014).

19. Holley, M. T., Nagarajan, N., Danielson, C., Zorlutuna, P. & Park, K. Development and characterization of muscle-based actuators for self-stabilizing swimming biorobots. *Lab Chip* 16, 3473–3484 (2016).

20. Nawroth, J. C. *et al.* A tissue-engineered jellyfish with biomimetic propulsion. *Nat. Biotechnol.* 30, 792–797 (2012).

21. Kriegman, S., Blackiston, D., Levin, M. & Bongard, J. A scalable pipeline for designing reconfigurable organisms. *Proc. Natl. Acad. Sci. U. S. A.* 117, 1853–1859 (2020).

22. Costantini, M. *et al.* Microfluidic-enhanced 3D bioprinting of aligned myoblast-laden hydrogels leads to functionally organized myofibers in vitro and in vivo. *Biomaterials* 131, 98–110 (2017).

23. Merceron, T. K. *et al.* A 3D bioprinted complex structure for engineering the muscle-tendon unit. *Biofabrication* 7, (2015).

24. Kang, H. W. *et al.* A 3D bioprinting system to produce human-scale tissue constructs with structural integrity. *Nat. Biotechnol.* 34, 312–319 (2016).

25. Noor, N. *et al.* 3D Printing of Personalized Thick and Perfusable Cardiac Patches and Hearts. *Adv. Sci.* 6, (2019).
