# Supplementary figures and images for: Recent trends in bioartificial muscle engineering and their applications in cultured meat, biorobotic systems and biohybrid implants
Source: Commun Biol. 2022 Jul 22;5:737. doi: 10.1038/s42003-022-03593-5 (PMC9307618; doi:10.1038/s42003-022-03593-5)

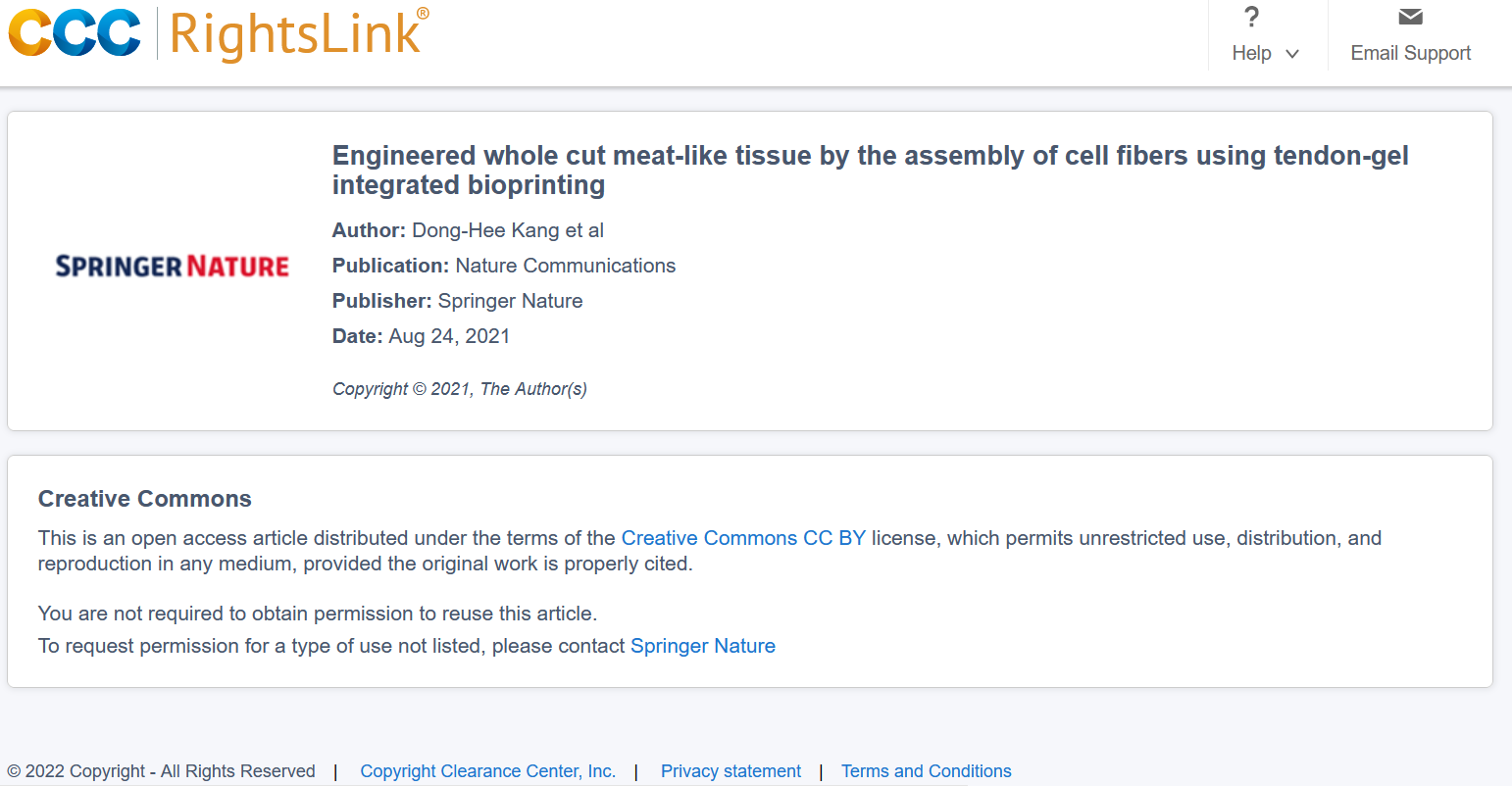

Supplement: Supplementary file 1 — Proof of Permissions [file 42003_2022_3593_MOESM1_ESM.zip › File 1 Kang 2021.png]

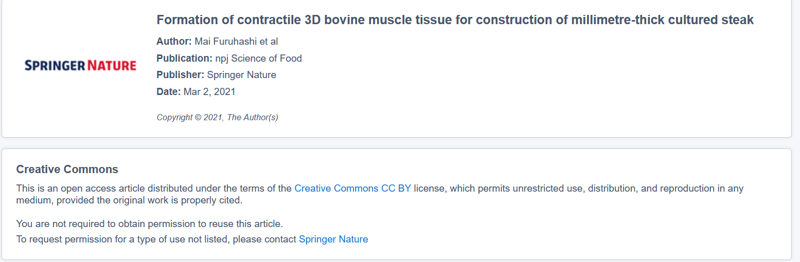

Supplement: Supplementary file 1 — Proof of Permissions [file 42003_2022_3593_MOESM1_ESM.zip › File 12 Furuhashi 2021.png]

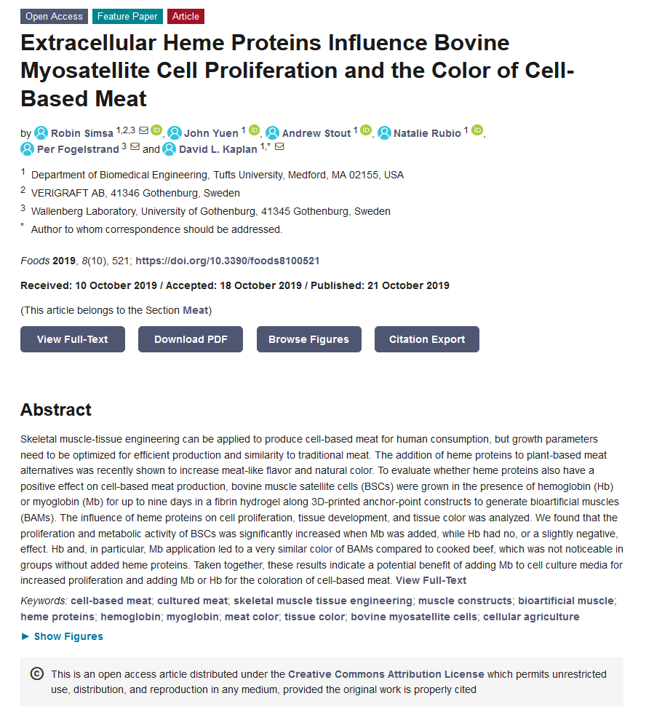

Supplement: Supplementary file 1 — Proof of Permissions [file 42003_2022_3593_MOESM1_ESM.zip › File 13 Simsa 2019.png]

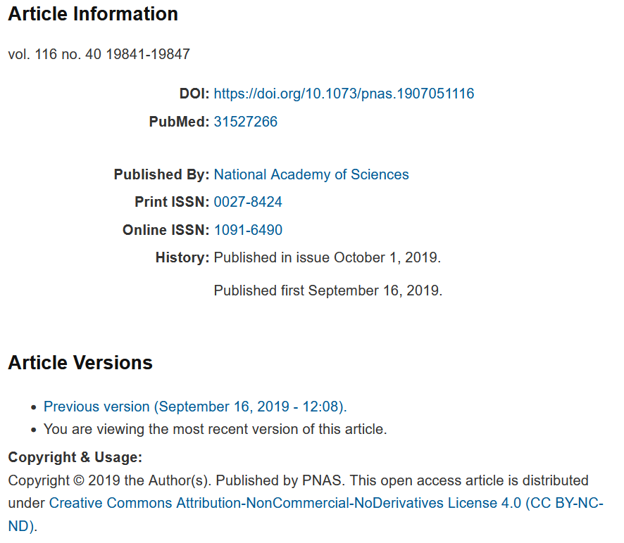

Supplement: Supplementary file 1 — Proof of Permissions [file 42003_2022_3593_MOESM1_ESM.zip › File 17 Aydin 2019.png]

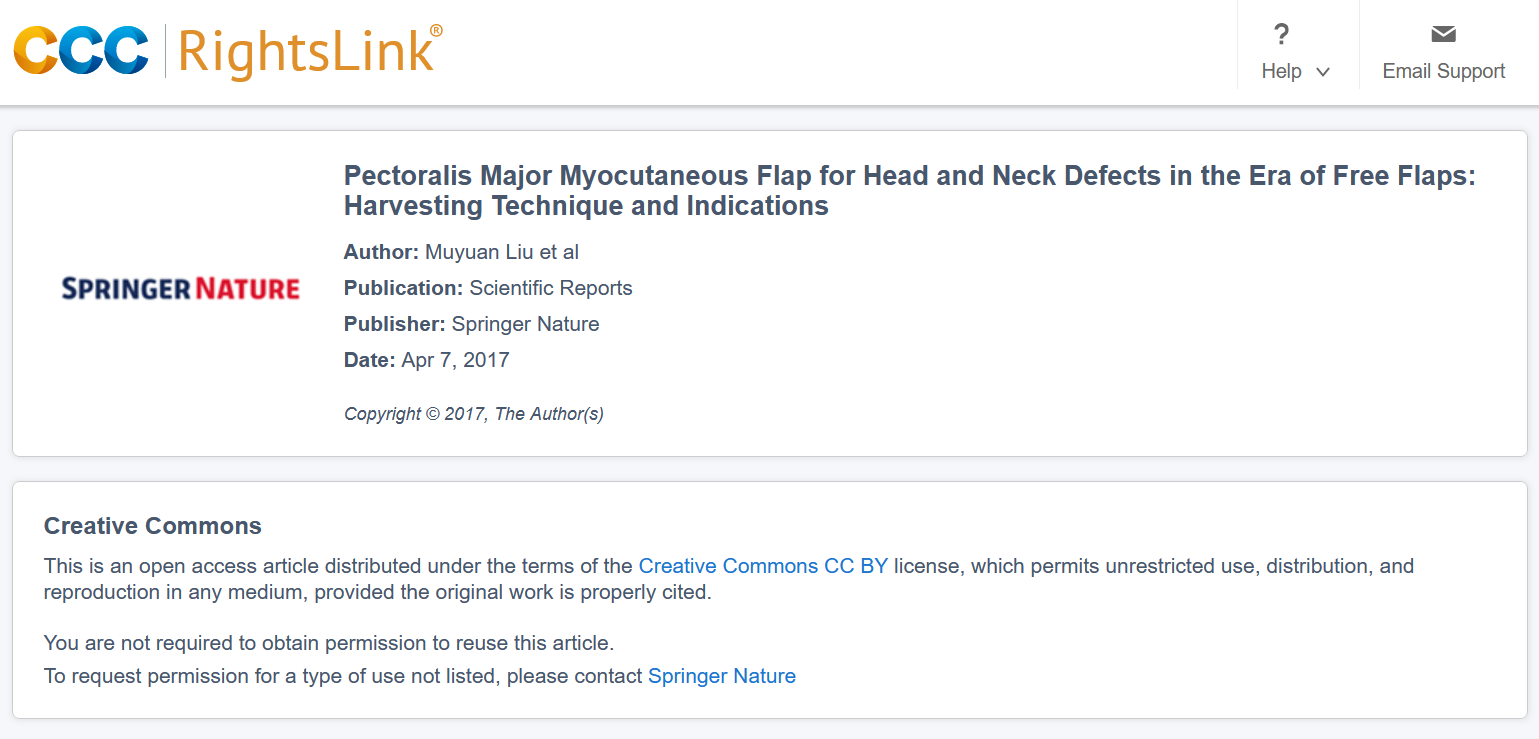

Supplement: Supplementary file 1 — Proof of Permissions [file 42003_2022_3593_MOESM1_ESM.zip › File 2 Liu 2017.png]

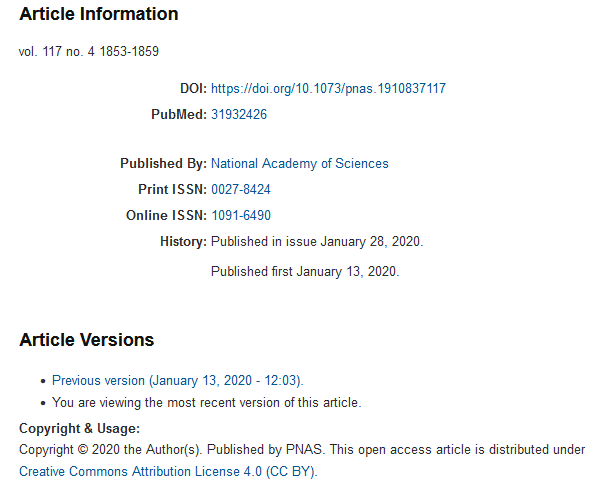

Supplement: Supplementary file 1 — Proof of Permissions [file 42003_2022_3593_MOESM1_ESM.zip › File 21 Kriegman 2020.png]

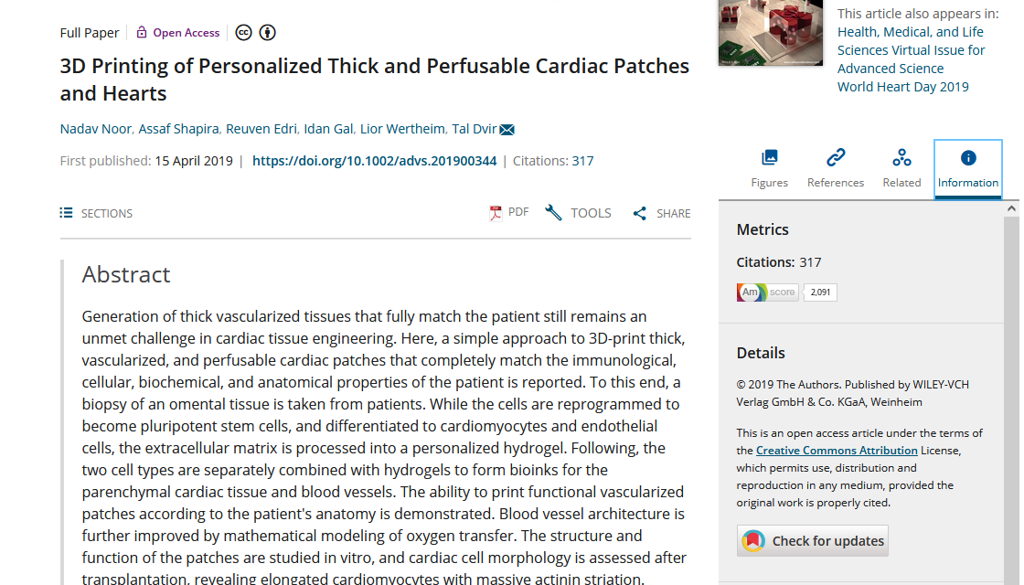

Supplement: Supplementary file 1 — Proof of Permissions [file 42003_2022_3593_MOESM1_ESM.zip › File 25 Noor 2019.png]

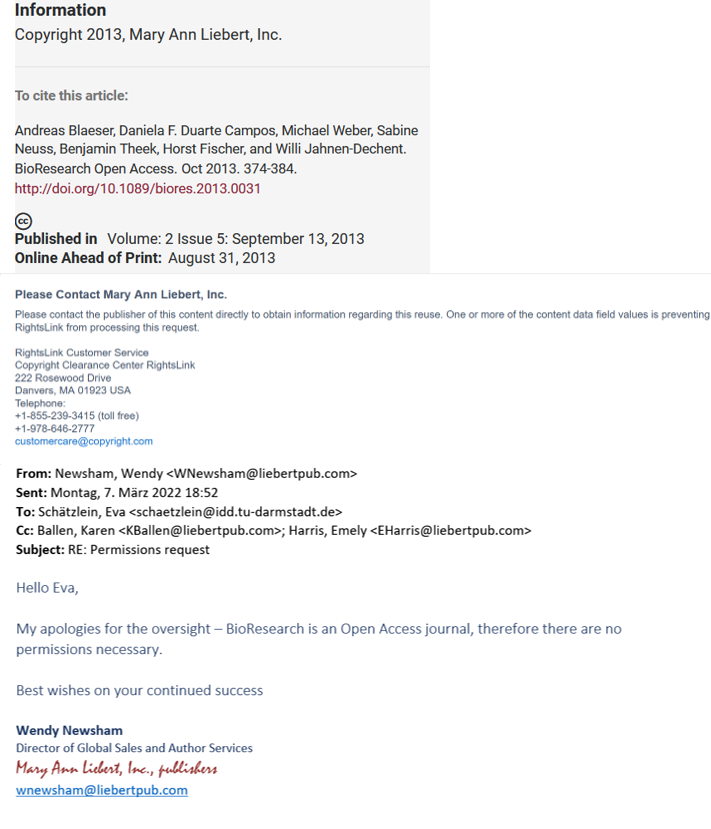

Supplement: Supplementary file 1 — Proof of Permissions [file 42003_2022_3593_MOESM1_ESM.zip › File 3 Blaeser 2013.png]

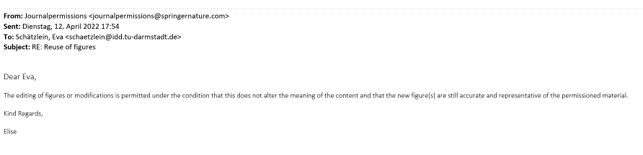

Supplement: Supplementary file 1 — Proof of Permissions [file 42003_2022_3593_MOESM1_ESM.zip › File 4 Srinivasan 2021_Modifications.png]

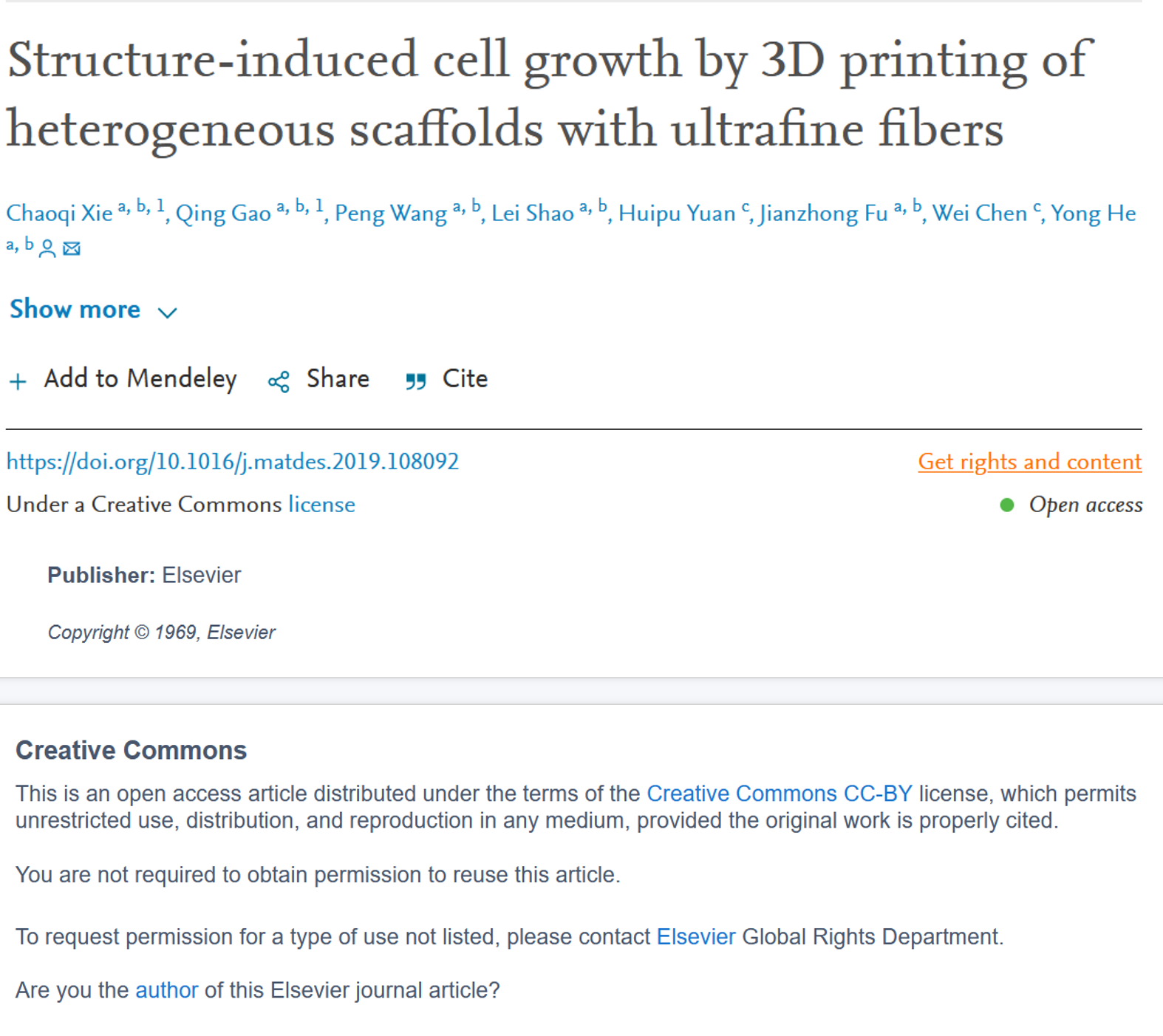

Supplement: Supplementary file 1 — Proof of Permissions [file 42003_2022_3593_MOESM1_ESM.zip › File 6 Xie 2019.png]

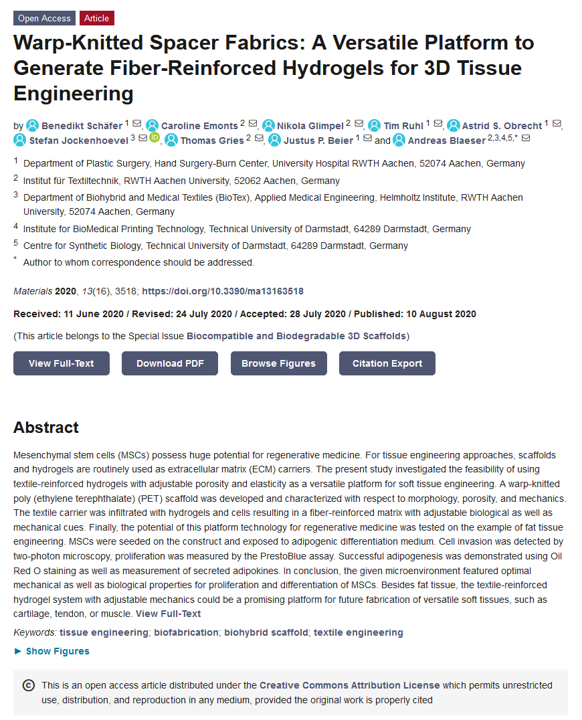

Supplement: Supplementary file 1 — Proof of Permissions [file 42003_2022_3593_MOESM1_ESM.zip › File 7 Schäfer 2020.png]

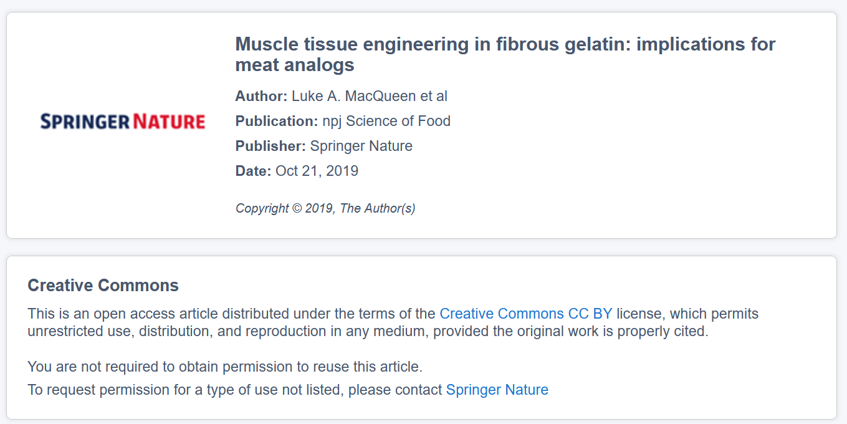

Supplement: Supplementary file 1 — Proof of Permissions [file 42003_2022_3593_MOESM1_ESM.zip › File 8 MacQueen 2019.png]
