# Supplementary material for: Recent trends in bioartificial muscle engineering and their applications in cultured meat, biorobotic systems and biohybrid implants
Source: Commun Biol. 2022 Jul 22;5:737. doi: 10.1038/s42003-022-03593-5 (PMC9307618; doi:10.1038/s42003-022-03593-5)
Supplement: Supplementary file 1 — Proof of Permissions [file File 15 Raman 2016.pdf › File 15 Raman 2016.pdf]

Schätzlein, Eva

From: PNAS Permissions <PNASPermissions@nas.edu>
Sent: Mittwoch, 2. März 2022 21:50
To: Schätzlein, Eva
Subject: RE: Permissions request

Dear Dr. Schätzlein,

Thank you for your message. Permission is granted for your use of the material as described in your request. Please include a complete citation for the original PNAS article when reusing the material. Because this material published after 2008, a copyright note is not needed. There is no charge for this material, either. Let us know if you have any questions.

Best regards,
Greg McCoy for
Diane Sullenberger
PNAS Executive Editor

From: Schätzlein, Eva <schaetzlein@idd.tu-darmstadt.de>
Sent: Monday, February 28, 2022 9:07 AM
To: PNAS Permissions <PNASPermissions@nas.edu>
Subject: Permissions request

Dear Sir or Madam,

I am preparing a review article with the name "Recent trends in bioartificial muscle engineering and their applications in cultured meat, biorobotic systems and biohybrid implants" to be published in the journal Communications Biology.

In order to finalize the article I am working on, I am requesting permission to use the material from an article published in your journal as described in detail below. Would you kindly grant permission use it?

Information about the requestor and the material:

Your full name, affiliation, and title	Eva Schätzlein, Technical University of Darmstadt, Institute for BioMedical Printing Technology, Darmstadt, Germany
Your complete mailing address, phone number, and email	Magdalenenstr. 2, 64289 Darmstadt, +4961511623776, schaetzlein@idd.tu-darmstadt.de
PNAS volume number, issue number, and issue date	PNAS March 29, 2016 113 (13); first published March 14, 2016;
PNAS article title	Optogenetic skeletal muscle-powered adaptive biological machines
PNAS authors' names	Ritu Raman, Caroline Cvetkovic, Sebastien G. M. Uzel, Randall J. Platt, Parijat Sengupta, Roger D. Kamm, and Rashid Bashir
Page numbers of items to be reprinted	3497-3502
Figure/table number or portion of text to be reprinted	Figure 1, Figure 3

Information about the intended use of the material:

Title of work in which PNAS material will appear	Recent trends in bioartificial muscle engineering and their applications in cultured meat, biorobotic systems and biohybrid implants
Authors/editors of work	Eva Schätzlein, Andreas Blaeser
Publisher of work	Springer Nature, Communications Biology
Retail price of work	Work will be published Open Access
Number of copies of work to be produced	1000+
Intended audience	professionals and beginners in the field of muscle biofabrication
Whether work is for nonprofit or commercial use	Work will be published Open Access

Thank you very much for your assistance! I am looking forward to your response.

Best regards,
Eva Schätzlein

Eva Schätzlein, M.Sc.
Research assistant,
BioMedical Printing Technology

Technical University of Darmstadt
Institute of Printing Science and Technology
Magdalenenstr. 2 (S1|10), Room 313
64289 Darmstadt

+49 (0) 6151 16-23776
eva.schaetzlein@tu-darmstadt.de
www.idd.tu-darmstadt.de
